# Development of a Peptide Inhibitor Targeting the C‐SH2 Domain of the SHP2 Phosphatase

**DOI:** 10.1002/cbic.202400938

**Published:** 2025-05-16

**Authors:** Azin Kiani, Catia L. Pierotti, Franziska Schedel, Thomas Kokot, Judith Weyershaeuser, Mario Brehm, Pablo Rios, Kerstin Fehrenbach, Bettina Warscheid, Susana Minguet, Wolfgang W. Schamel, Maja Köhn

**Affiliations:** ^1^ Signalling Research Centres BIOSS and CIBSS University of Freiburg Schänzlestraße 18 79104 Freiburg im Breisgau Germany; ^2^ Faculty of Chemistry and Pharmacy Hermann‐Staudinger Graduate School University of Freiburg Hebelstraße 27 79087 Freiburg im Breisgau Germany; ^3^ Institute of Biology III Faculty of Biology University of Freiburg Schänzlestraße 1 79104 Freiburg im Breisgau Germany; ^4^ Institute for Cell Biology Department of Molecular Cell Biology University of Bonn Käthe‐Kümmel‐Straße 1 53115 Bonn Germany; ^5^ Spemann Graduate School of Biology and Medicine University of Freiburg Albertstraße 19A 79104 Freiburg im Breisgau Germany; ^6^ Biochemistry II Theodor‐Boveri‐Institute University of Würzburg Am Hubland 97074 Würzburg Germany; ^7^ Centre of Chronic Immunodeficiency CCI University Clinics and Medical Faculty Breisacher Straße 115 79106 Freiburg im Breisgau Germany

**Keywords:** peptide inhibitors, phosphatases, pTyr mimetics, SH2 domains, SHP2

## Abstract

Src homology 2 (SH2) domain‐containing phosphatase 2 (SHP2) mediates important signal transduction upon cell surface receptor stimulation, regulating multiple cellular functions. In addition to the catalytically active phosphotyrosine (pTyr) phosphatase domain, SHP2 contains two regulatory pTyr‐binding domains: the N‐SH2 and C‐SH2 domains. While the role of the N‐SH2 domain is well understood, the role of the C‐SH2 domain is less clear. To support studies on the involvement of the domains in SHP2 function, herein, the development of a peptide inhibitor containing a nonhydrolysable pTyr mimetic, which selectively binds to the C‐SH2 domain of SHP2 and blocks its protein–protein interactions, is described. Incorporation of the pTyr mimetic l‐O‐malonyltyrosine (l‐OMT) results in robust binding affinity to the C‐SH2 domain, while the widely used pTyr mimetic phosphonodifluoromethyl phenylalanine (F_2_Pmp) abolishes binding, showing that this mimetic is not a general binder of SH2 domains, which challenges existing notions. The C‐SH2 inhibitor peptide (CSIP) is stable, selective, cell permeable, and noncytotoxic. CSIP enriches the toolbox of inhibitors with different modes of action targeting SHP2, and will support studies to better understand SHP2 regulation and interactions, which can ultimately inform new drug discovery efforts.

## Graphical abstract

A peptide inhibitor targeting the Src homology 2 (SH2) domain‐containing phosphatase 2 (SHP2) has been developed that binds to and blocks the C‐SH2 domain. The C‐SH2 inhibitor peptide is stable, selective, cell permeable, and noncytotoxic making it an enabling chemical tool to study SHP2 in cells.

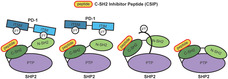

## 1 Introduction

**Figure 1:**
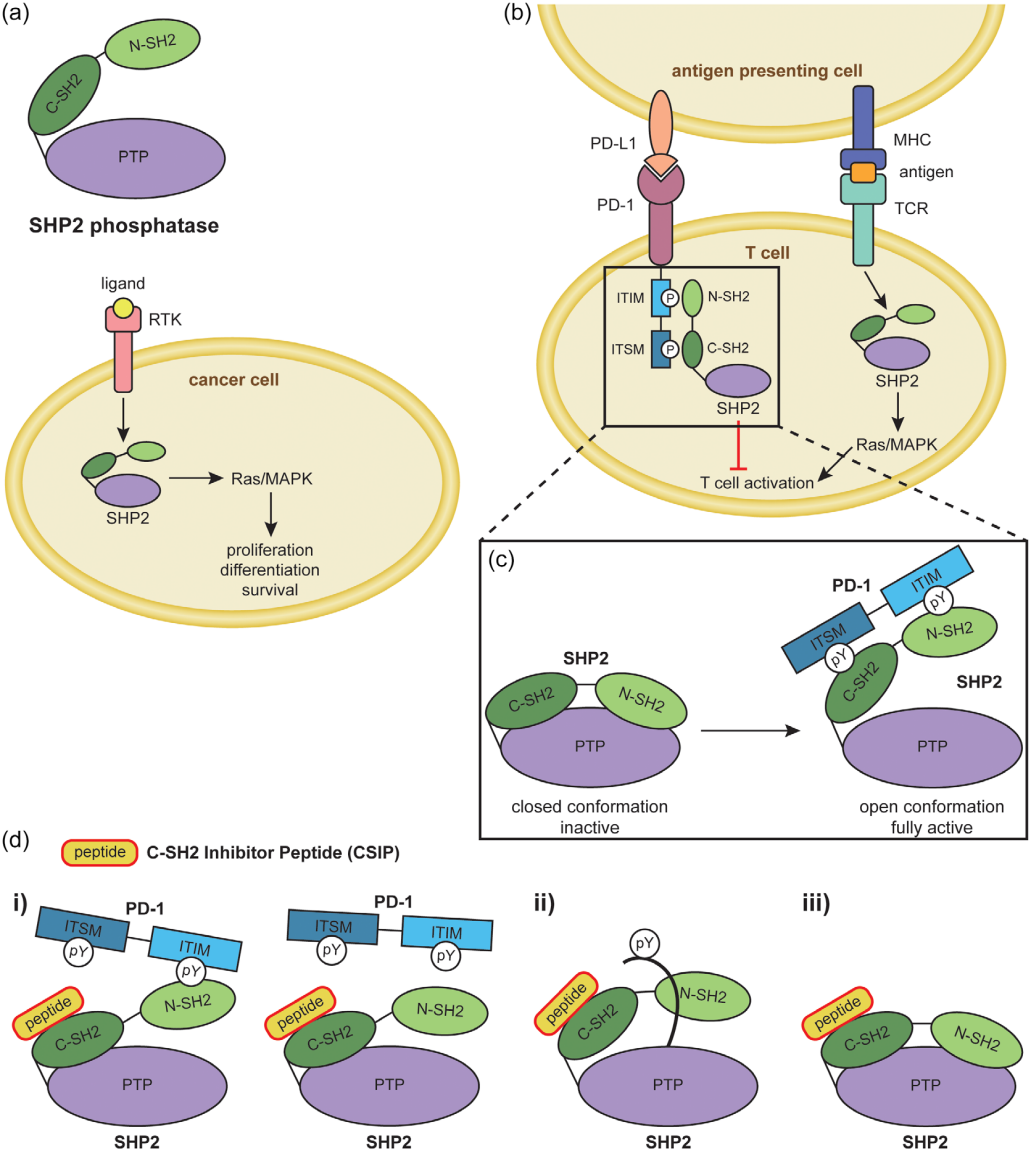
Overview of the SHP2 phosphatase, its role in cell signaling and possible C‐SH2 domain inhibition effects. a) SHP2 consists of three‐folded domains (N‐SH2, C‐SH2, and PTP) and is a key regulator downstream of RTKs that activates Ras/MAPK signaling, a pathway often dysregulated in cancer cells. b) In T cells, SHP2 is both a positive regulator of T cell activation downstream of the TCR and a negative regulator of T cell activation downstream of the inhibitory receptor PD‐1. c) SHP2 activation mechanism by the PD‐1 bisphosphoryl tyrosine‐based activation motif (BTAM) including ITIM and ITSM. In the basal state, SHP2 is autoinhibited by the N‐SH2 domain binding to and occluding the PTP domain active site. The binding of the PD‐1 ITIM and ITSM pTyr residues to the SHP2 N‐SH2 and C‐SH2 domains causes a conformational change that releases the PTP domain from the constraint of the N‐SH2 domain and activates SHP2 by trapping it in a stable open conformation. d) Selectively blocking the SHP2 C‐SH2 domain using a peptide inhibitor (C‐SH2 inhibitor peptide; CSIP) could lead to several different outcomes i) disruption of BTAM binding, ii) disruption of *C*‐terminal pTyr binding, or iii) silent binding.

Src homology 2 (SH2) domain‐containing phosphatase 2 (SHP2, PTPN11 gene) is a protein tyrosine phosphatase (PTP) and is a key regulator of signaling downstream of receptor tyrosine kinases.^[^ ^1^ ^]^ It activates the Ras/mitogen‐activated protein kinase (MAPK) pathway following receptor stimulation to promote cell proliferation, differentiation, and survival (**Figure** **1**a).^[^ ^2^ ^]^ The role of SHP2 in mediating aberrant receptor tyrosine kinase signaling in cancer and its function as a proto‐oncogene has led to the development of SHP2 inhibitors such as SHP099.^[^ ^3^, ^4^ ^]^ SHP099 fully inhibits SHP2 by locking SHP2 in an inactive conformation. Clinical trials with SHP2 inhibitors for oncology indications are currently ongoing.^[^ ^5^ ^]^ Furthermore, SHP2 has important functions in immune cells. For example, in addition to being a positive regulator of signaling downstream of the T cell receptor (TCR) via the Ras/MAPK pathway to promote T cell activation (Figure 1b),^[^ ^6^ ^]^ SHP2 is also a negative regulator of T cell activation via the inhibitory receptor programmed cell death protein 1 (PD‐1) (Figure 1b).^[^ ^7^, ^8^, ^9^ ^]^

Structurally, SHP2 consists of two SH2 domains (N‐SH2 and C‐SH2), a PTP domain and a *C*‐terminal tail (Figure 1a).^[^ ^10^ ^]^ In the absence of phosphotyrosine (pTyr) binding partners, direct intramolecular interactions between the N‐SH2 and PTP domains keep SHP2 in a closed, autoinhibited, and catalytically inactive conformation.^[^ ^10^ ^]^ Whereas the binding of a pTyr motif to the N‐SH2 domain is required for SHP2 activation, the role of the C‐SH2 domain is, in general, not as clearly understood. In the context of two adjacent pTyr‐containing motifs (together forming a bisphosphoryl tyrosine‐based activation motif, BTAM), found in proteins such as PD‐1, GRB2‐associated‐binding protein 1 (GAB1), and insulin receptor substrate 1 (IRS‐1), the binding of both pTyr to both SH2 domains in a 1:1 molecular stoichiometry enables full SHP2 activation (Figure 1c).^[^ ^11^, ^12^, ^13^, ^14^ ^]^ Functionally, this full activation of SHP2 leads to the activation of the Ras/MAPK pathway in the case of GAB1, but to the negative regulation of insulin and PD‐1 signaling.^[^ ^11^, ^12^, ^13^ ^]^ Another activation mechanism involving the *C*‐terminal tail can occur upon *C*‐terminal phosphorylation of SHP2, which induces intramolecular binding of the phosphorylated residue with the N‐SH2 or C‐SH2 domain.^[^ ^15^ ^]^ Given the intricate nature of the regulation of SHP2 through its SH2 domains, dissecting which mechanism is involved in a certain cellular response is a challenging endeavor.

So far, functional studies of the SHP2 SH2 domains rely on mutational alteration of either the SH2 domains or their pTyr binding partners. Such mutational alterations require the expression of protein variants in the cell line of interest, which could lead to cellular rewiring or cellular adaptation and therefore does not always reflect what occurs endogenously. Cell‐active, selective chemical inhibitors of the protein‐protein interactions of the SH2 domains enable the investigation of their function with spatiotemporal control involving endogenous proteins.^[^ ^16^ ^]^ In addition to the allosteric inhibitor SHP099, active site inhibitors^[^ ^17^, ^18^ ^]^ and a peptide inhibitor of the N‐SH2 domain,^[^ ^19^ ^]^ which is not yet cell‐permeable, have been developed. A C‐SH2 domain inhibitor would complement the SHP2 chemical inhibitor toolbox and aid in dissecting the roles of the C‐SH2 domain in different cellular processes.

To address this limitation, here, we developed a peptide that targets the C‐SH2 domain of SHP2 and blocks its interactions. Interestingly, blocking the C‐SH2 domain could lead to several different outcomes: i) impairing the full activation of SHP2 by preventing the binding of one or both pTyr motifs of the BTAM; ii) blocking the intramolecular interaction with the SHP2 *C*‐terminal pTyr or with other pTyr‐mediated interactions; and iii) a silent binding event, in case the C‐SH2 domain is not involved in the activation of SHP2 downstream of a given cell surface receptor or SHP2 is in its basal state (Figure 1d). Here, we show that our peptide, called CSIP, indeed inhibits the full activation of SHP2 in vitro triggered by the BTAM of PD‐1, which are the immunoreceptor tyrosine‐based inhibitory motif (ITIM) and immunoreceptor tyrosine‐based switch motif (ITSM).^[^ ^8^, ^9^, ^11^, ^20^ ^]^ CSIP contains a nonhydrolysable pTyr mimetic and is stable, selective, cell permeable and noncytotoxic. Of note, we observed a difference in the binding affinity of commonly used nonhydrolysable pTyr mimetics for the SHP2 SH2 domains,^[^ ^21^ ^]^ importantly including the unexpected complete loss of binding of the commonly used pTyr mimetic phosphonodifluoromethyl phenylalanine (F_2_Pmp). CSIP will enable the exploratory investigation of SHP2 function and regulation through its C‐SH2 domain in cellular contexts.

## 2 Results and Discussion

### 2.1 Development of a SHP2 C‐SH2 Inhibitor Peptide

**Figure 2:**
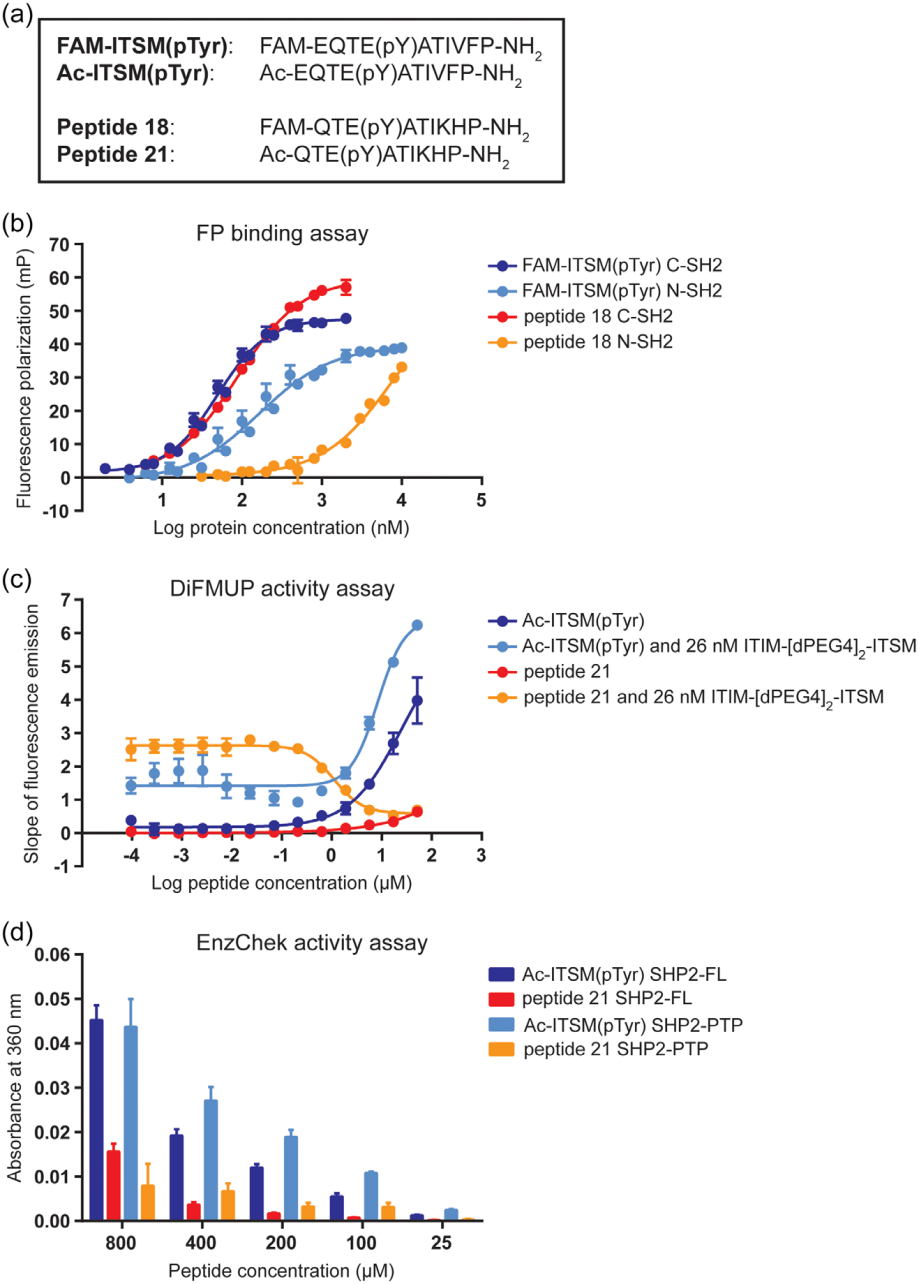
Development of a SHP2 C‐SH2 inhibitor peptide. a) Amino acid sequence of PD‐1 ITSM and derivative inhibitor peptides 18 and 21. 5(6)‐carboxyfluorescein (FAM)‐labeled peptides and acetylated (Ac) peptides were synthesized for binding assays and activity assays, respectively. b) Fluorescence polarization (FP) assay measuring the binding affinity between increasing concentrations of the SHP2 C‐SH2 domain and 100 nm FAM‐ITSM(pTyr) (blue) or peptide 18 (red), or increasing concentrations of the SHP2 N‐SH2 domain and 100 nm FAM‐ITSM(pTyr) (light blue) or peptide 18 (orange). Data represent the mean of three independent experiments, each performed in technical triplicates, and error bars represent SEM. c) DiFMUP activity assay evaluating the ability of four different conditions to activate or inhibit 0.5 nm full‐length SHP2: increasing concentrations of Ac‐ITSM(pTyr) (blue), 26 nm bisphosphorylated tandem ITIM‐[dPEG4]_2_‐ITSM peptide with increasing concentrations of Ac‐ITSM(pTyr) (light blue), increasing concentrations of peptide 21 (red), or 26 nm bisphosphorylated tandem ITIM‐[dPEG4]_2_‐ITSM peptide with increasing concentrations of peptide 21 (orange). Data represent the mean of three independent experiments, each performed in technical triplicates, and error bars represent SEM. d) EnzChek activity assay evaluating the ability of 50 nm full‐length (FL) SHP2 to dephosphorylate increasing concentrations of Ac‐ITSM(pTyr) (blue) or peptide 21 (red), or 50 nm isolated PTP domain to dephosphorylate increasing concentrations of Ac‐ITSM(pTyr) (light blue) or peptide 21 (orange). Data represent the mean of three independent experiments, each performed in technical triplicates, and error bars represent SEM.

In our previous work, we investigated the interaction between the PD‐1 ITIM/ITSM and the SHP2 N‐SH2/C‐SH2 domains.^[^ ^11^ ^]^ Given our previous findings that the PD‐1 ITSM has a strong binding affinity (in the nanomolar range) for both the N‐SH2 and C‐SH2 domains, with a preference for the C‐SH2 over the N‐SH2, the development of a peptide inhibitor of the SHP2 C‐SH2 domain was based on the ITSM sequence of PD‐1. To this end, a fluorescence polarization (FP) assay for screening the binding of peptides to both SH2 domains was established. First, the 5(6)‐carboxyfluorescein (FAM)‐labeled sequence of the PD‐1 ITSM was synthesized (**Figure** **2**a and S2a, Supporting Information), and its binding affinity for the recombinant SHP2 SH2 domains was measured by FP. FAM‐ITSM(pTyr) bound to the N‐SH2 and C‐SH2 domains with binding affinities (*K*_D_) of 164.0 and 48.9 nm, respectively (**Table** **1**, Figure 2b), which is in the same range as the *K*_D_ values of 105.9 and 58.8 nm obtained from isothermal titration calorimetry (ITC) (Table 1), similar to the previously reported ITC *K*_D_ values of 167 and 13 nm. ^[^ ^11^ ^]^ This supports the validity of the FP measurements for SH2‐domain binder screening.

**Table 1:** Key peptides generated that target the C‐SH2 domain of SHP2.

Peptide number/Namea)	Modification (*N*‐terminus)b)	Sequence	*K*_D_ ± SEM [nm]c)	IC_50_ ± SEM [μm]d)
Ac‐ITSM(pTyr)	Ac‐	EQTE(pY)ATIVFP‐NH_2_	C‐SH2: 58.8 (ITC) N‐SH2: 105.9 (ITC)	–
FAM‐ITSM(pTyr)	FAM‐	EQTE(pY)ATIVFP‐NH_2_	C‐SH2: 48.88 ± 3.15 N‐SH2: 164.0 ± 19.5	–
18	FAM‐	‐QTE(pY)ATIKHP‐NH_2_	C‐SH2: 88.16 ± 4.29 N‐SH2: >3000	–
21	Ac‐	‐QTE(pY)ATIKHP‐NH_2_	–	1.054 ± 0.502
22	Ac‐	‐QTE(F_2_Pmp)ATIKHP‐NH_2_	–	>51.20
23	Ac‐	‐QTE(Pmp)ATIKHP‐NH_2_	–	No inhibition
24	Ac‐	‐QTE(l‐OMT)ATIKHP‐NH_2_	–	7.816 ± 1.948
25	FAM‐	‐QTE(F_2_Pmp)ATIKHP‐NH_2_	C‐SH2: no binding N‐SH2: no binding	–
26	FAM‐	‐QTE(l‐OMT)ATIKHP‐NH_2_	C‐SH2: 1599 ± 388 N‐SH2: >32,000	–
27	–	C‐dPEG_2_‐QTE(l‐OMT)ATIKHP‐NH_2_	–	–
28 (FAM‐CSIP)e)	FAM‐	rrrrrrrr‐dPEG_2_‐QTE(l‐OMT)ATIKHP‐NH_2_	–	–
29 (Ac‐CSIP)e)	Ac‐	rrrrrrrr‐dPEG_2_‐QTE(l‐OMT)ATIKHP‐NH_2_	–	10.45 ± 4.17
FAM‐ITSM(F_2_Pmp)	FAM‐	EQTE(F_2_Pmp)ATIVFP‐NH_2_	C‐SH2: >2000 N‐SH2: 1394 ± 63	–
FAM‐ITSM(Pmp)	FAM‐	EQTE(Pmp)ATIVFP‐NH_2_	C‐SH2: >2000 N‐SH2: >10,000	–
FAM‐ITSM(l‐OMT)	FAM‐	EQTE(l‐OMT)ATIVFP‐NH_2_	C‐SH2: 257.2 ± 8.3 N‐SH2: 1714 ± 105	–

a) The sequence, binding affinity (K_D_), and in vitro activity (IC_50_) of key SHP2 C‐SH2 targeting peptides used in this study;

b) At the *N*‐terminus, peptides were either 5(6)‐carboxyfluorescein (FAM)‐labeled for FP binding assays, or acetylated (Ac) for DiFMUP activity assays or ITC binding assays;

c) *K*_D_ values obtained from FP binding assays are from three independent experiments, each performed in technical triplicates. ITC K_D_ values are from one experiment;

d) IC_50_ values obtained from DiFMUP activity assays are from two or three independent experiments, each performed in technical triplicates;

e) The optimized C‐SH2 inhibitor peptide is denoted as CSIP.

Our overarching aim was to identify substitutions in the ITSM sequence that resulted in similar or stronger binding to the C‐SH2 domain and weaker binding to the N‐SH2 domain. Thus, to determine the contribution of specific residues in the ITSM sequence for binding to the SH2 domains, a series of FAM‐labeled peptides based on the ITSM sequence that contain single Ala substitutions were synthesized and subjected to the FP binding assay (Table S1, Supporting Information). The *C*‐terminal Pro was kept intact as a structural anchor in proximity to Val203 of the C‐SH2 domain in the nuclear magnetic resonance structure 6R5G.^[^ ^11^ ^]^ The amino acid exchange *N*‐terminal of the pTyr did not improve the distinction between binding to the N‐SH2 and C‐SH2 domains (Table S1, Supporting Information). *C*‐terminally of the pTyr, replacement of the +3 Ile with Ala led to a loss of binding to both domains, whereas positions +4 and +5 were identified as strong determinators of binding selectivity between the N‐SH2 and C‐SH2 domains, albeit in both cases with reduced affinity. Subsequently, the available structures of the ITSM peptide bound to the C‐SH2 and N‐SH2 domains (PDB ID: 6R5G and 6ROZ, respectively; Figure S1a,b, Supporting Information)^[^ ^11^ ^]^ were investigated regarding differences concerning peptide binding, in particular relating to positions +4 and +5. To this end, it was apparent that the Val in +4 and Phe in +5 are close to Lys89 and Lys91 in the N‐SH2 domain, but proximal to Thr205 and Glu204 in the C‐SH2 domain. Therefore, to increase the binding affinity toward the C‐SH2 domain, the amino acids in positions +4 and +5 of the peptide were replaced by different positively charged amino acids. In addition, alternative nonpolar amino acids were tested as replacements for the present nonpolar *C*‐terminal amino acids, and the requirement for Glu in position −4 was tested, as the presence of negative charges often impairs peptide uptake.^[^ ^22^ ^]^ Accordingly, another series of FAM‐labeled peptides was synthesized and their binding affinities were measured by FP (Table 1 and S1, Supporting Information). From this peptide library, peptide 18 (Figure 2a and S2b, Supporting Information) with a *K*_D_ of 88.2 nm toward the C‐SH2 domain and excellent selectivity over the N‐SH2 domain (Figure 2b), was selected and further developed as an inhibitor of the SHP2 C‐SH2 domain. Our optimized sequence of peptide 18 is in agreement, regarding the amino acid properties preference of the C‐SH2 domain, with a recent study that described the high‐throughput profiling of SH2 ligand specificities using bacterial peptide display.^[^ ^23^ ^]^

To evaluate the activity of the C‐SH2 targeting peptide 18 in vitro, an acetylated version, peptide 21, was synthesized for testing in enzymatic assays (Figure 2a and S2b, Supporting Information). In these assays, the fluorogenic substrate 6,8‐difluoro‐4‐methylumbiliferyl phosphate (DiFMUP) was used to detect SHP2 (at 0.5 nm) phosphatase activity. As SHP2 is inactive in its basal state, 26 nm bisphosphorylated ITIM‐[dPEG4]_2_‐ITSM tandem peptide was added to partially activate SHP2, meaning that the SHP2 SH2 domains are either bound in a bidentate manner to the bisphosphorylated tandem peptide or not yet saturated.^[^ ^11^ ^]^ Under these conditions, it would be possible to either further activate SHP2 by binding to an unoccupied N‐SH2 domain, or to inhibit SHP2 by disrupting the bidentate binding of the tandem peptide at the C‐SH2 domain. To this set‐up, increasing concentrations of either Ac‐ITSM(pTyr) or peptide 21 were added (Figure 2c). The phosphatase activity of SHP2 increased with Ac‐ITSM(pTyr) alone or with the combination of Ac‐ITSM(pTyr) and the bisphosphorylated tandem peptide, as expected due to their possible interaction with the N‐SH2 domain. As we previously reported, the bisphosphorylated tandem peptide can bind to both SH2 domains simultaneously, while ITSM can bind to both the N‐SH2 and C‐SH2 domains individually.^[^ ^11^ ^]^ Thereby, the engagement of the N‐SH2 domain by the Ac‐ITSM(pTyr) peptide disrupts the closed autoinhibited conformation and promotes the formation of the open active state, which results in the observed increase in phosphatase activity. It is likely that the Ac‐ITSM(pTyr) peptide can also disrupt the bidentate binding of the tandem peptide to some extent, which could explain the unstable baseline at low Ac‐ITSM(pTyr) concentrations. In contrast to Ac‐ITSM(pTyr), the C‐SH2 targeting peptide 21 alone activated SHP2 only minimally at the highest peptide concentration. This is in agreement with the enzymatic data obtained when mutating the C‐SH2 domain to prevent peptide binding, showing that binding to the C‐SH2 domain alone has no effect on basal SHP2 activity in vitro.^[^ ^11^ ^]^ However, in the presence of the bisphosphorylated tandem peptide that activates SHP2, peptide 21 reduced SHP2 phosphatase activity with an IC_50_ of 1.05 μm. This demonstrates that peptide 21 prevents the tandem peptide from binding to both SH2 domains by blocking the C‐SH2 domain, thereby attenuating full SHP2 activation (Figure 2c). In this case, the N‐SH2 domain could still be bound to the bisphosphorylated tandem peptide, or the interaction could be completely lost (Figure 1d).

Peptide 21 contains a pTyr residue in its sequence and thus could be a potential substrate for the catalytic PTP domain, which could result in dephosphorylation of the peptide. Therefore, the suitability of peptide 21 as a substrate of SHP2 was evaluated in the EnzChek Phosphate Assay, which measures the release of inorganic phosphate from an enzymatic reaction, where either Ac‐ITSM(pTyr) or peptide 21 was added to full‐length SHP2 or the isolated PTP domain (Figure 2d). Full‐length SHP2 showed lower phosphatase activity than the PTP domain at concentrations of up to 400 μm Ac‐ITSM(pTyr) or peptide 21, due to the basal autoinhibition of full‐length SHP2. Overall, less dephosphorylation of peptide 21 than of Ac‐ITSM(pTyr) was observed with both full‐length SHP2 and the PTP domain. Moreover, dephosphorylation of peptide 21 by SHP2 required much higher concentrations than the nanomolar concentrations needed for binding to the C‐SH2 domain. Together, these data demonstrate that peptide 21 is not a good SHP2 substrate, and that its binding to the C‐SH2 domain requires much lower concentrations than the concentrations at which it is dephosphorylated, making peptide 21 a promising ligand targeting the C‐SH2 domain.

**Figure 3:**
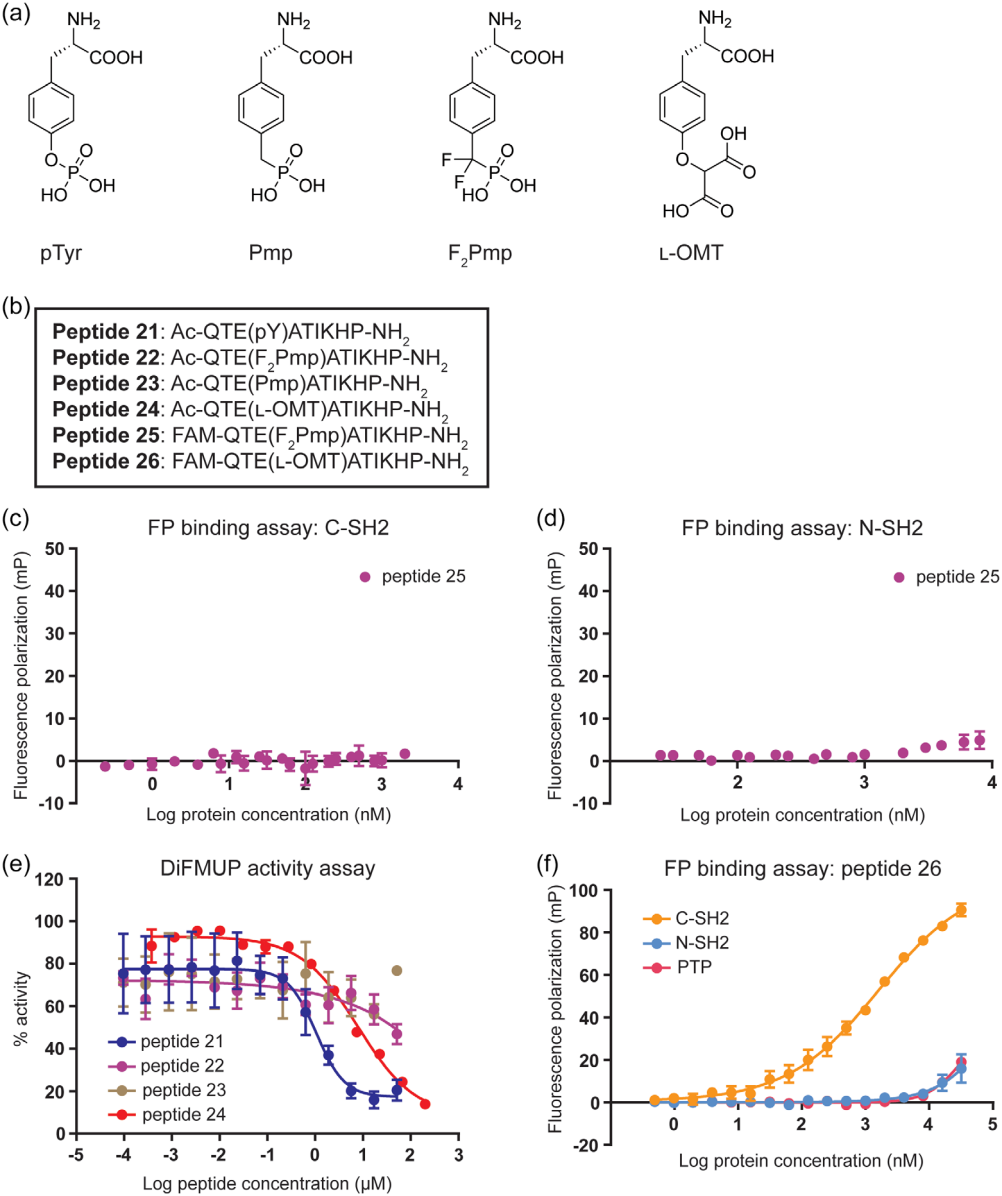
Incorporation of pTyr mimetics into the C‐SH2 inhibitor peptide. a) Chemical structure of pTyr and three nonhydrolysable pTyr mimetics: phosphonomethyl phenylalanine (Pmp), phosphonodifluoromethyl phenylalanine (F_2_Pmp), and l‐O‐malonyltyrosine (l‐OMT). b) Amino acid sequence of peptides 21, 22, 23, 24, 25, and 26 containing pTyr or the nonhydrolysable pTyr mimetics in (a). 5(6)‐carboxyfluorescein (FAM)‐labeled peptides and acetylated (Ac) peptides were synthesized for binding assays and activity assays, respectively. c) FP assay measuring the binding affinity between increasing concentrations of the SHP2 C‐SH2 domain and 100 nm peptide 25 containing the pTyr mimetic F_2_Pmp. Data represent the mean of three independent experiments, each performed in technical triplicates, and error bars represent SEM. d) FP assay measuring the binding affinity between increasing concentrations of the SHP2 N‐SH2 domain and 100 nm peptide 25 containing the pTyr mimetic F_2_Pmp. Data represent the mean of three independent experiments, each performed in technical triplicates, and error bars represent SEM. e) DiFMUP activity assay testing increasing concentrations of peptide 21 containing pTyr (blue), peptide 22 containing F_2_Pmp (purple), peptide 23 containing Pmp (light brown) or peptide 24 containing l‐OMT (red) for their ability to inhibit FL SHP2 (0.5 nm) activated with the tandem bisphosphorylated ITIM‐[dPEG4]_2_‐ITSM peptide (26 nm). Data represent the mean of two or three independent experiments, each performed in technical triplicates, and error bars represent SEM. f) FP assay measuring the binding affinity between 100 nm peptide 26 containing l‐OMT and increasing concentrations of the C‐SH2 (orange), N‐SH2 (light blue), or PTP (pink) domains of SHP2. Data represent the mean of three independent experiments, each performed in technical triplicates, and error bars represent SEM.

While the pTyr residue in FAM‐labeled peptide 18 or acetylated peptide 21 is essential for interacting with its target, the SHP2 C‐SH2 domain, the cleavable phosphate group makes it liable for dephosphorylation inside cells and could potentially result in instability of the peptide. Therefore, it was essential to replace the pTyr residue with a nonhydrolysable mimetic (**Figure** **3**a). To this end, the pTyr residue in peptide 18/21 was replaced with three nonhydrolysable mimetics: phosphonomethyl phenylalanine (Pmp), phosphonodifluoromethyl phenylalanine (F_2_Pmp), or l‐O‐malonyltyrosine (l‐OMT). They were synthesized as either FAM‐labelled peptides, for binding assays, or acetylated peptides, for activity assays (Figure 3b). Introducing the commonly used pTyr mimetic F_2_Pmp into the peptide sequence (FAM‐labeled peptide 25) resulted in the complete loss of binding to both SHP2 SH2 domains (Figure 3c,d). This was particularly surprising for the stronger binding C‐SH2 domain, as the N‐SH2 domain was already disfavored by the peptide when carrying a pTyr (peptide 18). Consistently, incorporation of F_2_Pmp (acetylated peptide 22) or Pmp (acetylated peptide 23) resulted in the loss of inhibitory activity against SHP2 compared to peptide 21 (Figure 3e). Interestingly, substitution with l‐OMT (acetylated peptide 24; Figure S2c, Supporting Information) inhibited the activity of SHP2 with an IC_50_ of 7.82 μm (Figure 3e), which was only slightly weaker than peptide 21. Furthermore, l‐OMT incorporation (FAM‐labelled peptide 26; Figure S2c, Supporting Information) retained binding to the C‐SH2 domain, albeit with an increased *K*_D_ of 1.60 μm compared to peptide 18, and maintained binding selectivity for the C‐SH2 domain over the N‐SH2 and PTP domains (Figure 3f).

To corroborate the unexpected observation that the F_2_Pmp substituted peptide did not bind to the SHP2 SH2 domains, we synthesized and assessed FAM‐labeled ITSM peptides containing pTyr mimetics Pmp, F_2_Pmp, or l‐OMT in FP binding assays (Figure S3a, Supporting Information). When examining their binding to the C‐SH2 domain, we observed a marked decrease in binding affinity for both FAM‐ITSM(F_2_Pmp) and FAM‐ITSM(Pmp) (Figure S3b, Supporting Information). FAM‐ITSM(l‐OMT) retained binding to the C‐SH2 domain, albeit weaker than FAM‐ITSM(pTyr) (Figure S3b, Supporting Information). For the N‐SH2 domain, FAM‐ITSM(Pmp) showed greatly reduced binding, however, FAM‐ITSM(F_2_Pmp) showed slightly better binding than FAM‐ITSM(l‐OMT), although both weaker than FAM‐ITSM(pTyr) (Figure S3c, Supporting Information). Together, this confirms the preference of the C‐SH2 domain for l‐OMT as a pTyr mimetic and the loss of binding observed with F_2_Pmp.

In the structures of the ITSM peptide bound to the C‐SH2 and N‐SH2 domains (PDB ID: 6R5G and 6ROZ),^[^ ^11^ ^]^ there are key hydrogen bond (H‐bond) interactions between the phosphate oxygens of the ITSM pTyr and an Arg residue of the SH2 domain (R138 in the C‐SH2 domain and R32 in the N‐SH2 domain) (Figure S1a,b, Supporting Information). This Arg residue is crucial for the ability of the SH2 domains to bind phosphopeptides, as mutation of the Arg to Ala (R138A and R32A for the C‐SH2 and N‐SH2 domain, respectively) results in abrogation of SHP2 activation by ITSM, ITIM, or ITIM‐[dPEG4]_2_‐ITSM.^[^ ^11^ ^]^ In the structures, R32 forms one H‐bond with the phosphate ester oxygen and two with another phosphate oxygen, whereas R138 only forms two H‐bonds in total, of which one is with the phosphate ester oxygen. Therefore, the loss of binding and activity observed for the F_2_Pmp and Pmp substituted peptides could be due to the absence of the phosphate ester oxygen, resulting in these pTyr mimetics being unable to form a crucial hydrogen bond with the key Arg residues, impacting the C‐SH2 domain binding more than that of the N‐SH2 domain due to the fewer interactions formed by the C‐SH2 domain. As another possibility, a recent study suggested that different pTyr orientations are accessible when bound to the N‐SH2 domain.^[^ ^24^ ^]^ Perhaps binding to the C‐SH2 domain does not allow for different orientations, making the loss of the oxygen less tolerable than for the N‐SH2 domain. The l‐OMT substituted peptide, where an ester oxygen is preserved, would still be able to maintain these important interactions and this could explain the retained binding affinity and inhibitory activity observed. As a result, l‐OMT was selected as the nonhydrolysable pTyr mimetic to incorporate in the peptide targeting the SHP2 C‐SH2 domain (Figure S2c, Supporting Information).

### 2.2 SHP2 C‐SH2 Inhibitor Peptide is Stable, Selective, Cell Permeable, and Noncytotoxic

To evaluate the stability of the inhibitor peptide containing l‐OMT in cell lysate, FAM‐labeled peptide 26 was incubated with Jurkat T cell lysate for 24 h at 37 °C (Figure S4, Supporting Information). The relative amount of peptide 26, compared to FAM alone, was determined over the 24 h period by liquid chromatography‐mass spectrometry (LC‐MS). The LC‐MS analysis showed that peptide 26 was stable in Jurkat cell lysate over this time period.

**Figure 4:**
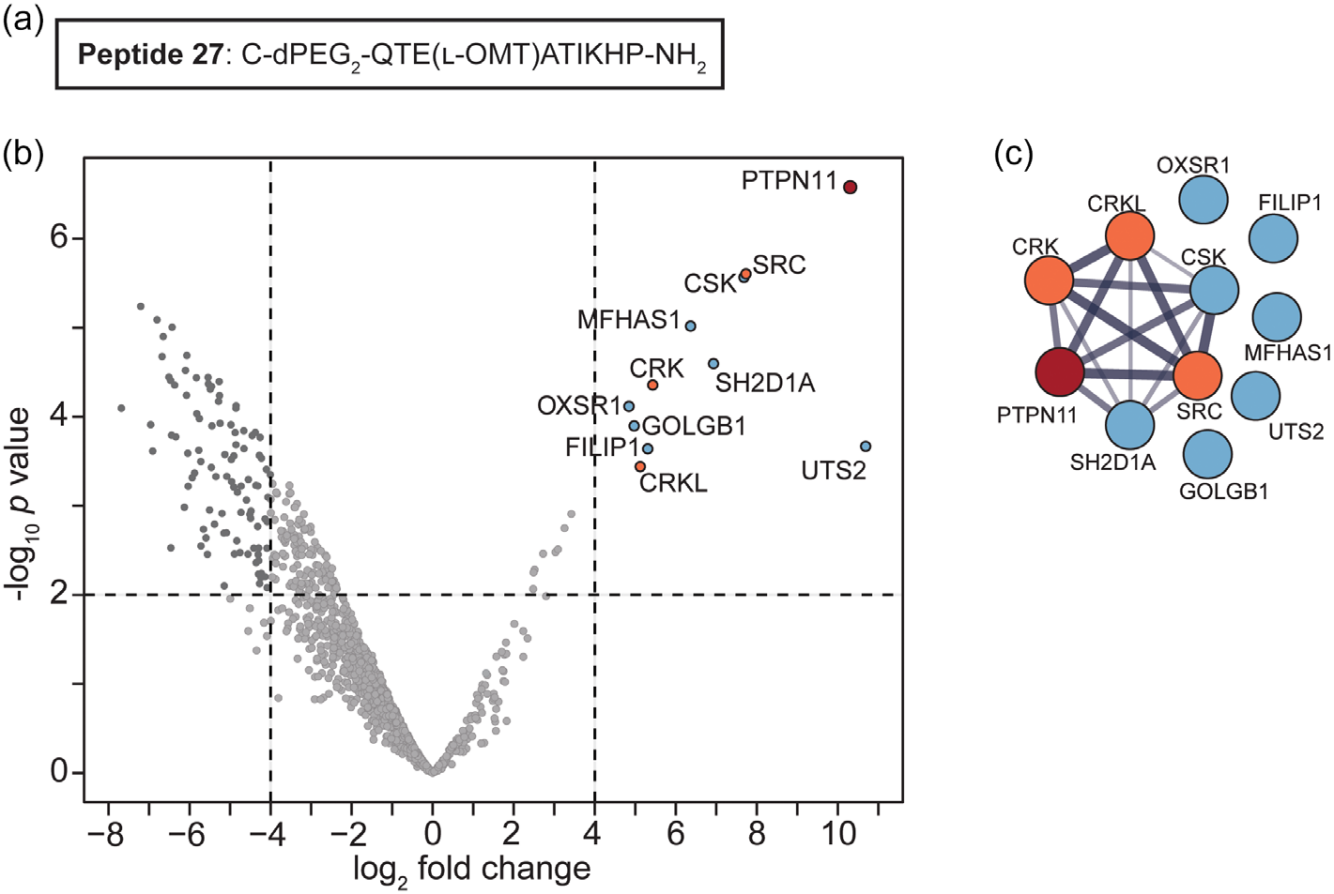
Selectivity of the C‐SH2 inhibitor peptide in Jurkat T cell lysate. a) Amino acid sequence of peptide 27 containing a Cys residue to enable immobilization to SulfoLink beads. b) Pulldown assay utilizing precoupled peptide 27 immobilized to SulfoLink beads and control beads not coupled to any peptide, performed with WT Jurkat T cell lysates and analyzed by mass spectrometry (MS). Proteins significantly enriched by peptide 27 beads are highlighted in blue, with SHP2 in red and SHP2 interactors in orange. Enrichment was assessed using a limma *t*‐test (*n* = 4), with a *p*‐value of 0.01. Dashed lines depict the thresholds for enrichment (*p*‐value of 0.01 and log_2_ fold change of 4). Proteins are annotated with their corresponding gene names. c) STRING network analysis of the 11 proteins that coenriched with peptide 27. Color coding is consistent with (b) and proteins are annotated with their corresponding gene names.

To investigate whether this peptide could bind specifically to endogenous SHP2, pulldown experiments were performed using a modified peptide attached to a solid resin. Peptide 24 containing l‐OMT was coupled to a dPEG_2_ linker and a Cys residue at the *N*‐terminus to generate peptide 27 (**Figure** **4**a). This modified peptide was then covalently immobilized to SulfoLink beads.^[^ ^25^ ^]^ SulfoLink beads that had not been coupled to any peptide were used as the negative control. In the pulldown experiments (*n* = 4), peptide 27 immobilized beads and negative control beads were incubated with Jurkat cell lysate overnight and the proteins enriched by the precoupled beads were analyzed by MS.

The acquired MS data were quantitatively analyzed, yielding eleven proteins significantly enriched with peptide 27 compared to the negative control (Figure 4b). Importantly, among these proteins, SHP2 (PTPN11) was the most prominent hit, while the closely related protein SHP1 (PTPN6) was absent (Table S2, Supporting Information), underscoring the selectivity of peptide 27 within the cell lysate. Additionally, the identified proteins underwent comparison with the BioGrid and DEPOD databases to discern known SHP2 interactors,^[^ ^26^ ^]^ among which Src, CRK, and CRKL were significantly coenriched within the peptide 27 fraction, suggesting their indirect co‐enrichment through SHP2. STRING network analysis revealed functional and physical protein associations among SHP2, Src, CRK, CRKL, SH2D1A, and CSK (Figure 4c), supporting their co‐occurrence.^[^ ^27^ ^]^ Of note, while UTS2 showed significance in the log_2_ fold change, UTS2 does not contain an SH2 domain and sequence coverage was below 6% (Table S2, Supporting Information) and was therefore not followed up upon.

*C*‐terminal Src kinase (CSK), which contains an SH2 domain, is a negative regulator of Src family kinases.^[^ ^28^ ^]^ It has been reported that SHP2 regulates the activity of Src by controlling the recruitment of CSK, which suggests a potential indirect binding mechanism.^[^ ^29^ ^]^ Nevertheless, we selected CSK for further investigation as a potential off‐target due to it being the most abundant hit not previously reported as a direct SHP2 interactor. To assess any potential direct binding of the inhibitor peptide to CSK, the binding affinity of FAM‐labeled peptide 26 (Figure S5a, Supporting Information) for the SH2 domain of CSK was measured by FP (Figure S5b, Supporting Information). Compared to the SHP2 C‐SH2 domain, the binding of peptide 26 to the CSK SH2 domain was at least two orders of magnitude weaker and only showed binding at the highest concentrations, demonstrating that the inhibitor peptide only very weakly binds to CSK in vitro. Thus, the enrichment of CSK in the pulldown likely arises from indirect interactions, as supported by the STRING network analysis.

Since Src was enriched to a similar extent as CSK in the pulldown and contains an SH2 domain, we also investigated Src as a potential off‐target of the inhibitor peptide. The binding of peptide 26 to the SH2 domain of Src was determined by FP (Figure S5c, Supporting Information). Compared to the binding affinity of peptide 26 for the SHP2 C‐SH2 domain, the binding affinity of peptide 26 for the Src SH2 domain was around1.5 orders of magnitude weaker, again corroborating the selectivity of the peptide for the C‐SH2 domain of SHP2.

**Figure 5:**
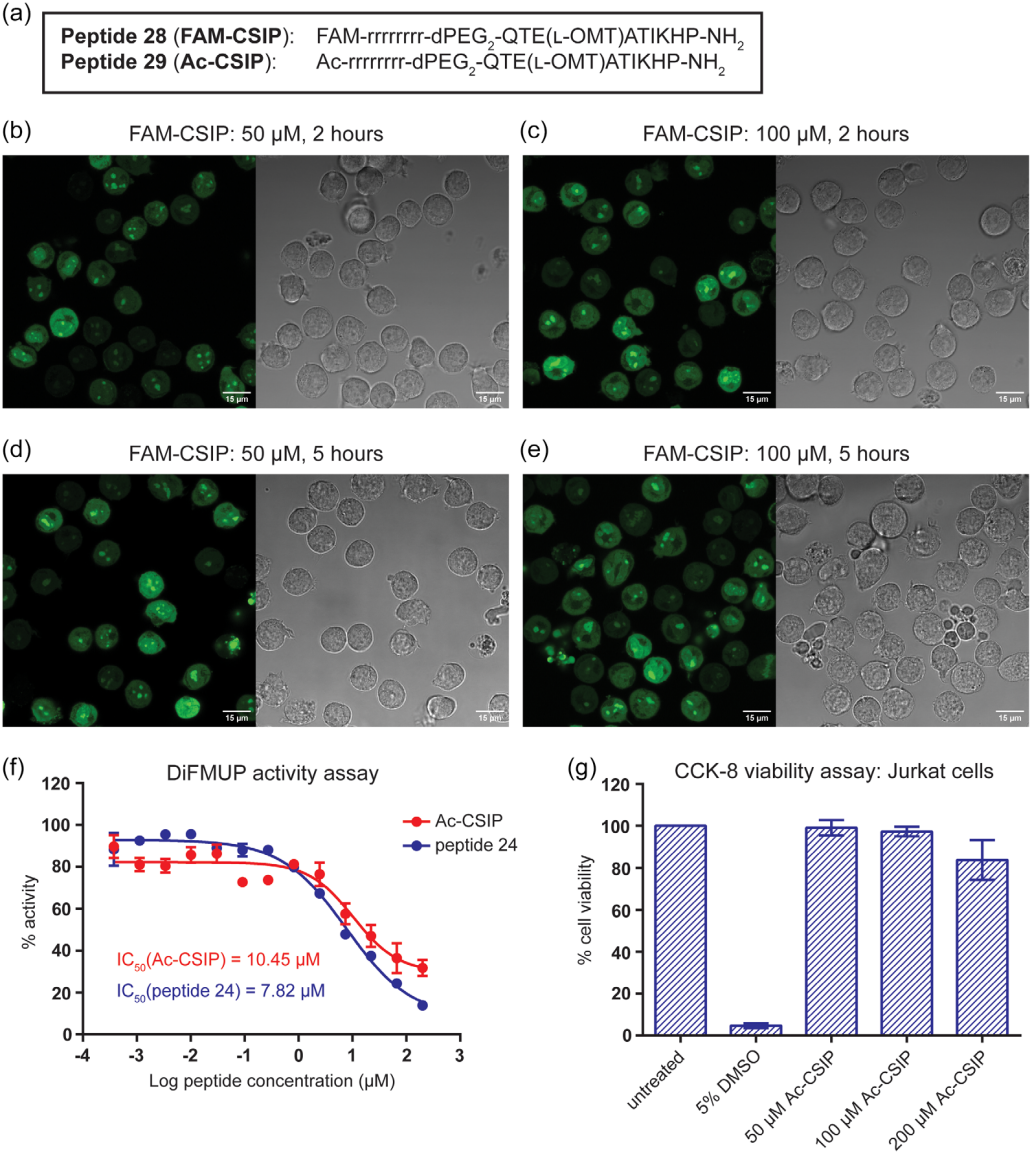
Cell permeability and cytotoxicity of the C‐SH2 inhibitor peptide in Jurkat T cells. a) Amino acid sequence of peptides 28 and 29 containing a poly dArg sequence for cell permeability, yielding the 5(6)‐carboxyfluorescein (FAM) and acetylated (Ac) derivatives of the optimized C‐SH2 inhibitor peptide (CSIP). b–e) WT Jurkat T cells were incubated with FAM‐CSIP (peptide 28) at the indicated concentrations and for the indicated time points. Cells were imaged by confocal fluorescence microscopy to visualize the cellular uptake of the peptide via its FAM group. Fluorescence (left) and transmission (right) images are shown and scale bars are 15 μM. Representative images are shown from three independent experiments. f) DiFMUP activity assay testing increasing concentrations of Ac‐CSIP (peptide 29; red) or peptide 24 (blue) for their ability to inhibit full‐length SHP2 (0.5 nm) activated with the tandem bisphosphorylated ITIM‐[dPEG4]_2_‐ITSM peptide (26 nm). Data represent the mean of two or three independent experiments, each performed in technical triplicates, and error bars represent SEM. g) CCK‐8 assay evaluating the cytotoxicity of 50–200 μm Ac‐CSIP (peptide 29) following 24 h incubation with WT Jurkat T cells. Vehicle (H_2_O) treated cells were used as the negative control and set to 100% viability, while cells treated with 5% DMSO to induce cell death were used as the positive control. Data represent the mean of four independent experiments, each performed in technical triplicates, and error bars represent SEM.

Next, we sought to investigate the cell permeability of the inhibitor peptide. The cellular uptake of FAM‐labeled peptide 26 in Jurkat cells was evaluated by confocal fluorescence microscopy, however, following incubation with 50 or 100 μm peptide for 2 or 5 h, no cell uptake was observed (except in dead cells or cell debris; Figure S6a, Supporting Information). To optimize the peptide for cell penetration, a poly dArg and dPEG_2_ linker were installed between the FAM group and the peptide 26 sequence, to generate FAM‐labeled peptide 28 (**Figure** **5**a and S2d, Supporting Information).^[^ ^30^ ^]^ Using the same live cell imaging experimental approach, peptide 28 showed robust cell permeability at both 50 and 100 μm following either 2 or 5 h incubation with the peptide (Figure 5b–e). To also test the cell uptake in a common cancer cell line, HeLa cells were treated with FAM‐labeled peptide 28. 100 μm peptide 28 after 30 min or 2 h showed good cell permeability in HeLa cells (Figure S6b, Supporting Information).

To ensure that the dArg sequence did not interfere with the activity, the corresponding acetylated peptide, peptide 29 (Figure 5a and S2d, Supporting Information), was synthesized and subjected to the DiFMUP assay and its activity was compared to that of peptide 24. Peptide 29 retained the ability to inhibit SHP2, with an IC_50_ of 10.45 μm, which was comparable to peptide 24 and thus ruled out any potential detrimental effects of the poly dArg and dPEG_2_ linker on the activity (Figure 5f). Therefore, peptide 28 and peptide 29 became our FAM and acetylated derivatives of the optimized C‐SH2 inhibitor peptide (CSIP) (see also Figure S2d, Supporting Information).

Finally, the cytotoxicity of CSIP was evaluated. The impact of CSIP on the viability of Jurkat cells and HeLa cells was investigated using the Cell Counting Kit‐8 (CCK‐8). Jurkat cells incubated with Ac‐CSIP (peptide 29) for 24 h only started showing a trend toward some toxicity at the highest concentration of 200 μm (Figure 5g), while HeLa cells incubated with Ac‐CSIP (peptide 29) for 16 h showed no toxicity even at a concentration of 300 μM (Figure S7, Supporting Information).

## 3 Conclusion

In this study, we developed a peptide ligand for the C‐SH2 domain, which blocks the C‐SH2 domain without disrupting the N‐SH2 domain and prevents SHP2 from reaching full activation triggered by the PD‐1 BTAM in vitro. The stability, selectivity, cell permeability, and cytotoxicity of the inhibitory peptide, CSIP, were established in cells. Overall, we demonstrated that CSIP is a fully characterized chemical tool targeting the SHP2 C‐SH2 domain. Future studies will involve determining the effect of targeting the C‐SH2 domain in signaling pathways downstream of various cell surface receptors, an area of research that is largely unexplored. As the role of the C‐SH2 domain in SHP2‐mediated signal transduction is generally less well understood than the N‐SH2 domain, CSIP is a valuable tool for investigating C‐SH2 domain function in cells.

An important finding of this work is that the nonhydrolysable pTyr mimetic F_2_Pmp was unable to bind to the SHP2 C‐SH2 domain, despite F_2_Pmp being widely used to mimic the characteristics of pTyr,^[^ ^21^, ^31^ ^]^ and as a chemical probe to enrich endogenous SH2 domain‐containing proteins.^[^ ^32^ ^]^ Incorporation of F_2_Pmp in the ITSM peptide or inhibitor peptide abolished binding to the C‐SH2 domain, while F_2_Pmp incorporation in the ITSM peptide resulted in weaker binding to the N‐SH2 domain and in the inhibitor peptide resulted in complete loss of binding to the N‐SH2 domain. This demonstrates that the application of pTyr mimetics is highly case‐dependent and using them as a general tool, in particular F_2_Pmp, should be approached with caution, as a general ability to bind SH2 domains cannot be assumed.

To conclude, CSIP is an enabling chemical tool for investigating the role of SHP2, in particular its C‐SH2 domain, in different cellular contexts. It enables the modulation of SHP2 function with precise temporal and reversible control, rather than permanently like genetic perturbations such as mutational or knockdown/knockout approaches. Along with the recently developed N‐SH2 inhibitor peptide,^[^ ^19^ ^]^ which would need to be further optimized to enable targeting the N‐SH2 domain of endogenous SHP2 in intact cells, and allosteric inhibitors such as SHP099,^[^ ^3^, ^4^ ^]^ as well as active site inhibitors,^[^ ^17^, ^18^ ^]^ our CSIP enriches the toolbox of chemical modulators of SHP2. This toolbox will be crucial for future research on understanding SHP2 function at a molecular level and will inform future SHP2 drug discovery efforts.

## Conflict of Interest

The authors declare no conflict of interest.

## Supporting information

Supplementary Material

## Data Availability

Mass spectrometry data are available at the ProteomeXchange Consortium (proteomecentral.proteomexchange.org) through the PRIDE partner repository under the following identifier PXD054302. All other raw data are available through the authors.
