# Supplementary material for: Development of a Peptide Inhibitor Targeting the C‐SH2 Domain of the SHP2 Phosphatase
Source: Chembiochem. 2025 May 16;26(10):e202400938. doi: 10.1002/cbic.202400938 (PMC12118337; doi:10.1002/cbic.202400938)
Supplement: Supplementary file 1 — Supplementary Material [file CBIC-26-e202400938-s001.zip › Kiani et al Supporting Information.pdf]

Supporting Information

Development of a Peptide Inhibitor Targeting the C-SH2 Domain of the SHP2 Phosphatase

Azin Kiani,[a][b][c]+ Catia L. Pierotti,[a][c][d]+ Franziska Schedel,[a][b][c][d][e] Thomas Kokot,[a][c] Judith Weyershaeuser,[a][c] Mario Brehm,[c] Pablo Rios,[a][c] Kerstin Fehrenbach,[a][c] Bettina Warscheid,[a][f] Susana Minguet,[a][c][g] Wolfgang W. Schamel,[a][c][g] and Maja Köhn[a][c][d]*

[a] Signalling Research Centres BIOSS and CIBSS, University of Freiburg, Schänzlestraße 18, 79104 Freiburg im Breisgau, Germany

[b] Faculty of Chemistry and Pharmacy, Hermann-Staudinger Graduate School, University of Freiburg, Hebelstraße 27, 79087 Freiburg im Breisgau, Germany

[c] Institute of Biology III, Faculty of Biology, University of Freiburg, Schänzlestraße 1, 79104 Freiburg im Breisgau, Germany

[d] Institute for Cell Biology, Department of Molecular Cell Biology, University of Bonn, Käthe-Kümmel-Straße 1, 53115 Bonn, Germany

[e] Spemann Graduate School of Biology and Medicine, University of Freiburg, Albertstraße 19A, 79104 Freiburg im Breisgau, Germany

[f] Biochemistry II, Theodor-Boveri-Institute, University of Würzburg, Am Hubland 97074 Würzburg, Germany

[g] Centre of Chronic Immunodeficiency CCI, University Clinics and Medical Faculty, Breisacher Straße 115, 79106 Freiburg im Breisgau, Germany

+ Co-first authors

* Corresponding author: mkoehn@uni-bonn.de

Table of Contents

Supporting Figures and Tables……………………………………………………………..3

Experimental Section..……..……………………………………………………………11

List of Abbreviations……………………………………………………………..11

Chemical Synthesis and Analytical Data of the Peptides………………………12

Biochemical Experiments…………………………………………………………30

Cellular Experiments……………………………………………………………37

References……………………………………………………………………………43

Supporting Figures and Tables

Table S1: Full list of peptides generated that target the C-SH2 domain of SHP2.

The sequence, molecular weight, binding affinity (K_D) and *in vitro* activity (IC_{50}) of all SHP2 C-SH2 targeting peptides synthesised in this study. The optimised C-SH2 inhibitor peptide is denoted as CSIP. Rows highlighted in grey indicate key peptides reported in **Table 1**. At the *N*-terminus, peptides were either 5(6)-carboxyfluorescein (FAM)-labelled for FP binding assays, or acetylated (Ac) for DiFMUP activity assays or ITC binding assays. K_D values obtained from FP binding assays are from three independent experiments, each performed in technical triplicates. ITC K_D values are from one experiment. IC_{50} values obtained from DiFMUP activity assays are from two or three independent experiments, each performed in technical triplicates.

**Peptide Number / Name**	**Modification (*N*-terminus)**	**Sequence**	**Calculated mass**	**Observed mass**	**K_D ± SEM (nM)**	**IC_{50} ± SEM (µM)**
Ac-ITSM(pTyr)	Ac-	EQTE(pY)ATIVFP-NH$_2$	1419.6	1419.6	C-SH2: 58.8 (ITC)<br>N-SH2: 105.9 (ITC)	
FAM-ITSM(pTyr)	FAM-	EQTE(pY)ATIVFP-NH$_2$	1735.9	1735.8	C-SH2: 48.88 ± 3.15<br>N-SH2: 164.0 ± 19.5	
1	FAM-	**A**QTE(pY)ATIVFP-NH$_2$	1677.9	1678.5	C-SH2: 62.03 ± 1.98<br>N-SH2: 295.4 ± 37.2	
2	FAM-	E**A**TE(pY)ATIVFP-NH$_2$	1677.9	1677.4	C-SH2: 51.29 ± 1.08<br>N-SH2: 57.96 ± 4.60	
3	FAM-	EQ**A**E(pY)ATIVFP-NH$_2$	1705.9	1706.5	C-SH2: 134.6 ± 4.6<br>N-SH2: 428.1 ± 25.1	
4	FAM-	EQT**A**(pY)ATIVFP-NH$_2$	1677.9	1678.5	C-SH2: 95.07 ± 14.38<br>N-SH2: 234.0 ± 17.6	
5	FAM-	EQTE(pY)A**A**IVFP-NH$_2$	1705.9	1706.5	C-SH2: 126.0 ± 6.2<br>N-SH2: 1140 ± 585	
6	FAM-	EQTE(pY)AT**A**VFP-NH$_2$	1692.8	1692.7	C-SH2: no binding<br>N-SH2: >10000	
7	FAM-	EQTE(pY)ATI**A**FP-NH$_2$	1705.8	1707.5	C-SH2: >2000<br>N-SH2: 1082 ± 84	
8	FAM-	EQTE(pY)ATIV**A**P-NH$_2$	1660.8	1660.4	C-SH2: 184.6 ± 30.8<br>N-SH2: >2000	
9	FAM-	EQTE(pY)ATI**K**FP-NH$_2$	1763.9	1763.6	C-SH2: 72.01 ± 2.61<br>N-SH2: >2000	
10	FAM-	EQTE(pY)AT**VK**FP-NH$_2$	1750.9	1751.4	C-SH2: 208.7 ± 14.4<br>N-SH2: >3000	
11	FAM-	-QTE(pY)ATIVFP-NH$_2$	1607.5	1607.5	C-SH2: 40.85 ± 0.96<br>N-SH2: 204.4 ± 10.4	
12	FAM-	-QTE(pY)ATI**KI**P-NH$_2$	1601.9	1602.5	C-SH2: 141.4 ± 6.2<br>N-SH2: not measured	
13	FAM-	-QTE(pY)ATI**KW**P-NH$_2$	1674.9	1675.4	C-SH2: 46.76 ± 1.29<br>N-SH2: 368.4 ± 40.0	
14	FAM-	-QTE(pY)A**V**I**K**FP-NH$_2$	1632.9	1634.6	C-SH2: 80.16 ± 4.32<br>N-SH2: 840.4 ± 228.4	
15	FAM-	-**K**TE(pY)ATI**K**FP-NH$_2$	1634.9	1636.4	C-SH2: 61.52 ± 1.83<br>N-SH2: 1548 ± 528	
16	FAM-	-QTE(pY)ATI**K**FP-NH$_2$	1635.8	1636.5	C-SH2: 86.00 ± 2.43<br>N-SH2: 1090 ± 199	
17	FAM-	-QTE(pY)ATI**KY**P-NH$_2$	1652.8	1652.3	C-SH2: 115.6 ± 7.7<br>N-SH2: >3000	
18	FAM-	-QTE(pY)ATI**KH**P-NH$_2$	1625.8	1626.5	C-SH2: 88.16 ± 4.29<br>N-SH2: >3000	
19	FAM-	-**K**TE(pY)ATI**KH**P-NH$_2$	1624.9	1625.4	C-SH2: 104.8 ± 3.6<br>N-SH2: not measured	
20	FAM-	-**KE**E(pY)ATI**KH**P-NH$_2$	1652.9	1654.7	C-SH2: >500<br>N-SH2: not measured	

21	Ac-	-QTE(pY)ATI**KH**P-NH_2	1308.4	1308.6		1.054 ± 0.502
22	Ac-	-QTE(**F_2Pmp**)ATI**KH**P-NH_2	1342.5	1342.6		>51.20
23	Ac-	-QTE(**Pmp**)ATI**KH**P-NH_2	1306.6	1306.5		No inhibition
24	Ac-	-QTE(**L-OMT**)ATI**KH**P-NH_2	1331.6	1332.5		7.816 ± 1.948
25	FAM-	-QTE(**F_2Pmp**)ATI**KH**P-NH_2	1659.9	1659.7	C-SH2: no binding<br>N-SH2: no binding	
26	FAM-	-QTE(**L-OMT**)ATI**KH**P-NH_2	1647.9	1647.5	C-SH2: 1599 ± 388<br>N-SH2: >32000	
27		C-dPEG$_2$-QTE(L-OMT)ATIKHP-NH_2	1335.8	1538.4		
28 (**FAM-CSIP**)	FAM-	rrrrrrrr-dPEG$_2$-QTE(L-OMT)ATIKHP-NH_2	3040.7	1520.9		
29 (**Ac-CSIP**)	Ac-	rrrrrrrr-dPEG$_2$- QTE(L-OMT)ATIKHP-NH_2	2723.7	909.1		10.45 ± 4.17
FAM-ITSM(F_2Pmp)	FAM-	EQTE(**F_2Pmp**)ATIVFP-NH_2	1767.7	885.0	C-SH2: >2000<br>N-SH2: 1394 ± 63	
FAM-ITSM(Pmp)	FAM-	EQTE(**Pmp**)ATIVFP-NH_2	1732.7	867.0	C-SH2: >2000<br>N-SH2: >10000	
FAM-ITSM(L-OMT)	FAM-	EQTE(**L-OMT**)ATIVFP-NH_2	1756.7	878.9	C-SH2: 257.2 ± 8.3<br>N-SH2: 1714 ± 105	

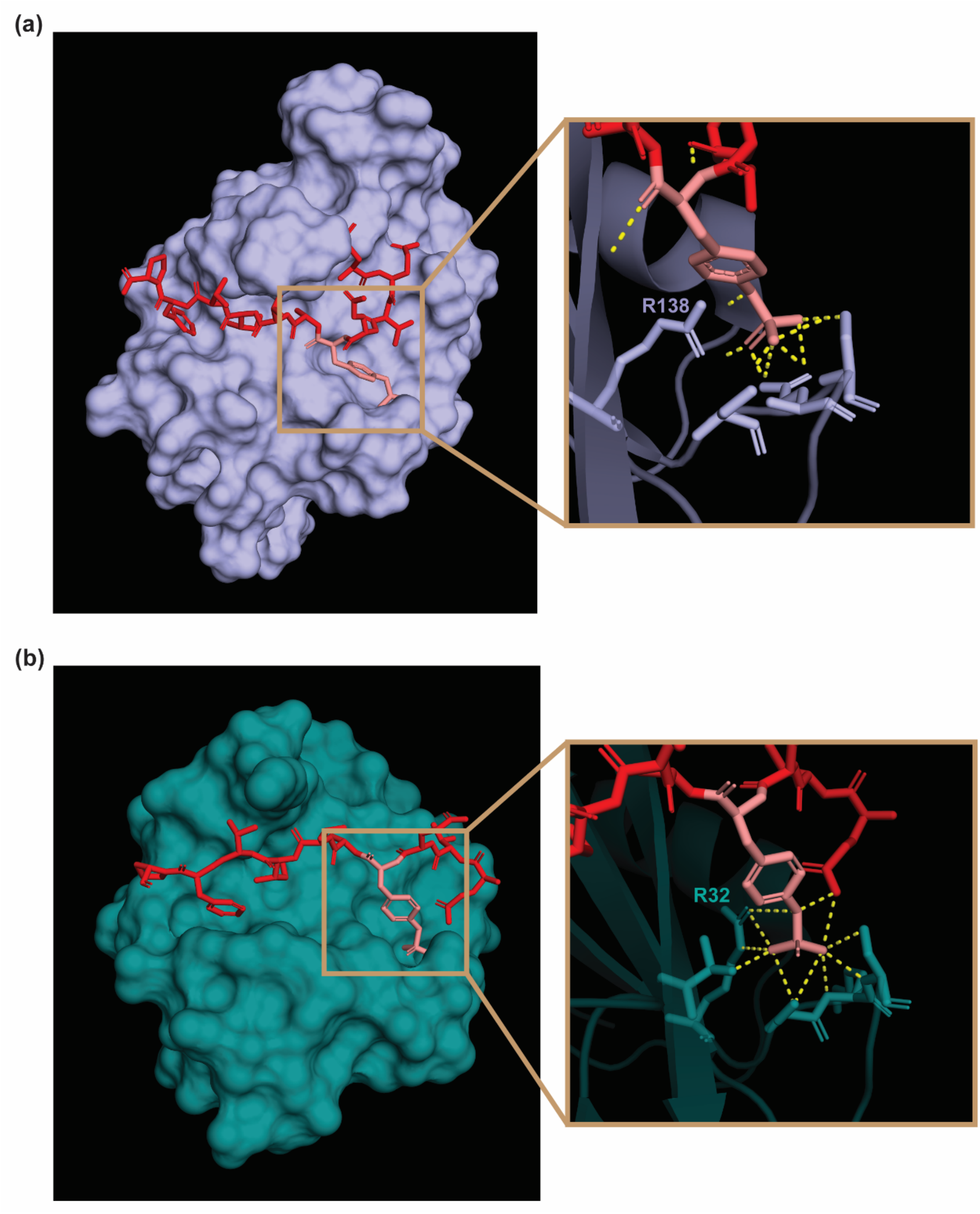

Figure S1: pTyr binding pocket of the SHP2 SH2 domains.

(a) NMR structure (PDB ID: 6R5G)[1] of the SHP2 C-SH2 domain (lilac) bound to the ITSM peptide (red), with a zoomed-in perspective of the pTyr binding pocket showing the interactions (yellow) between the pTyr (pink) and the C-SH2 domain (lilac). All interactions of the pTyr within a radius of 5 Å are displayed.
(b) X-ray crystal structure (PDB ID: 6ROZ)[1] of the SHP2 N-SH2 domain (cyan) bound to the ITSM peptide (red), with a zoomed-in perspective of the pTyr binding pocket showing the interactions (yellow) between the pTyr (pink) and the N-SH2 domain (cyan). All interactions of the pTyr within a radius of 5 Å are displayed.

(a)

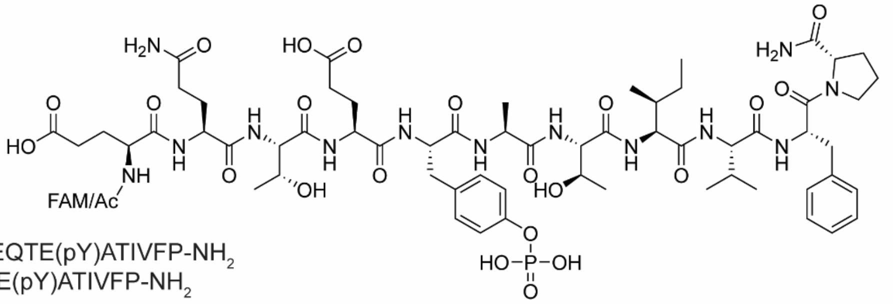

FAM-ITSM(pTyr): FAM-EQTE(pY)ATIVFP-NH_2
Ac-ITSM(pTyr): Ac-EQTE(pY)ATIVFP-NH_2

(b)

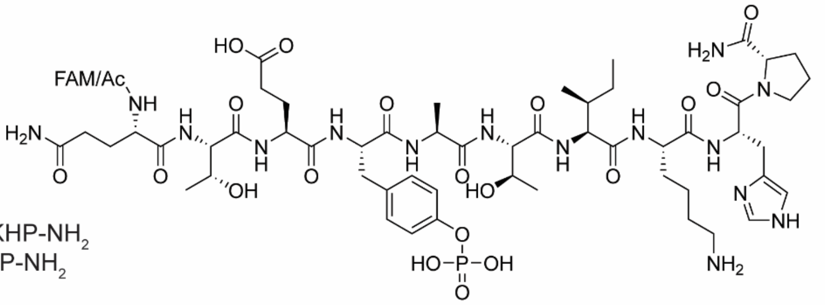

Peptide 18: FAM-QTE(pY)ATIKHP-NH_2
Peptide 21: Ac-QTE(pY)ATIKHP-NH_2

(c)

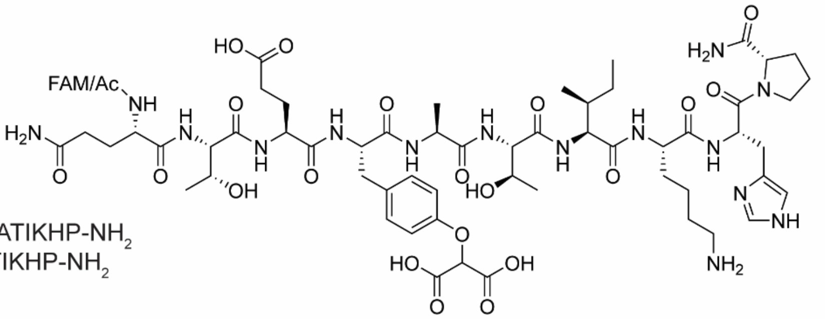

Peptide 26: FAM-QTE(ʟ-OMT)ATIKHP-NH_2
Peptide 24: Ac-QTE(ʟ-OMT)ATIKHP-NH_2

(d)

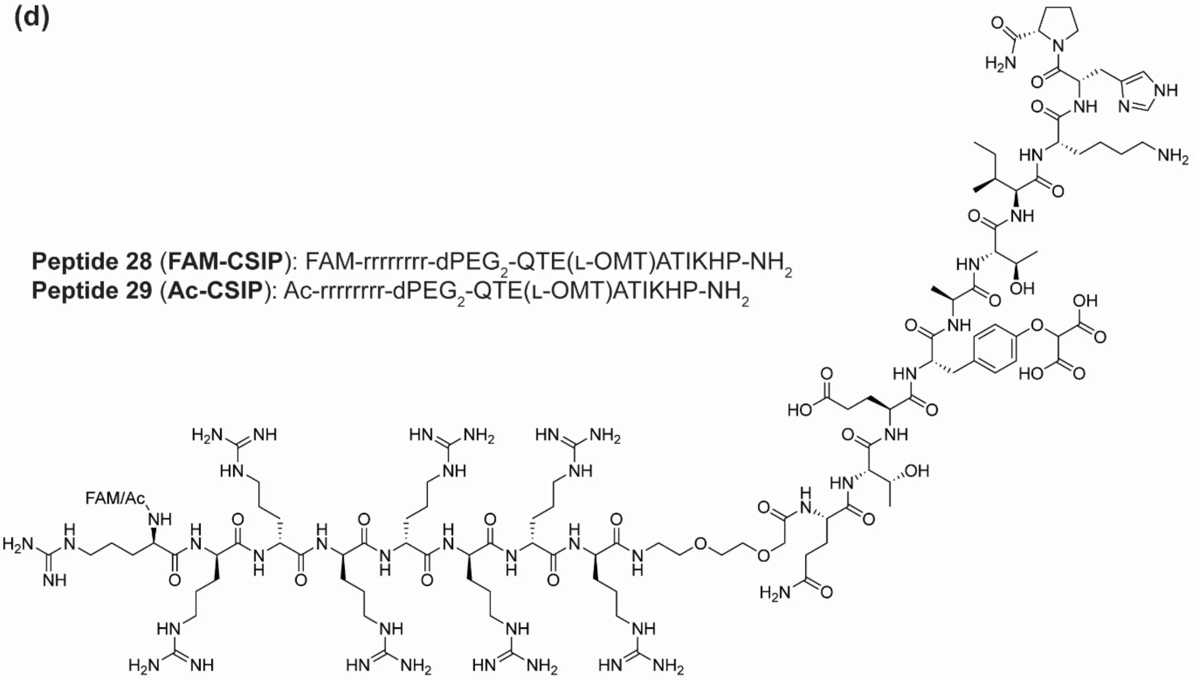

Peptide 28 (**FAM-CSIP**): FAM-rrrrrrrr-dPEG$_2$-QTE(ʟ-OMT)ATIKHP-NH_2
Peptide 29 (**Ac-CSIP**): Ac-rrrrrrrr-dPEG$_2$-QTE(ʟ-OMT)ATIKHP-NH_2

Figure S2: Key peptides targeting the C-SH2 domain of SHP2.

(a – d) Amino acid sequence and chemical structure of the key SHP2 C-SH2 targeting peptides synthesised in this study. 5(6)-carboxyfluorescein (FAM)-labelled peptides and acetylated (Ac) peptides were synthesised for binding assays and activity assays, respectively. The optimised C-SH2 inhibitor peptide is denoted as CSIP.

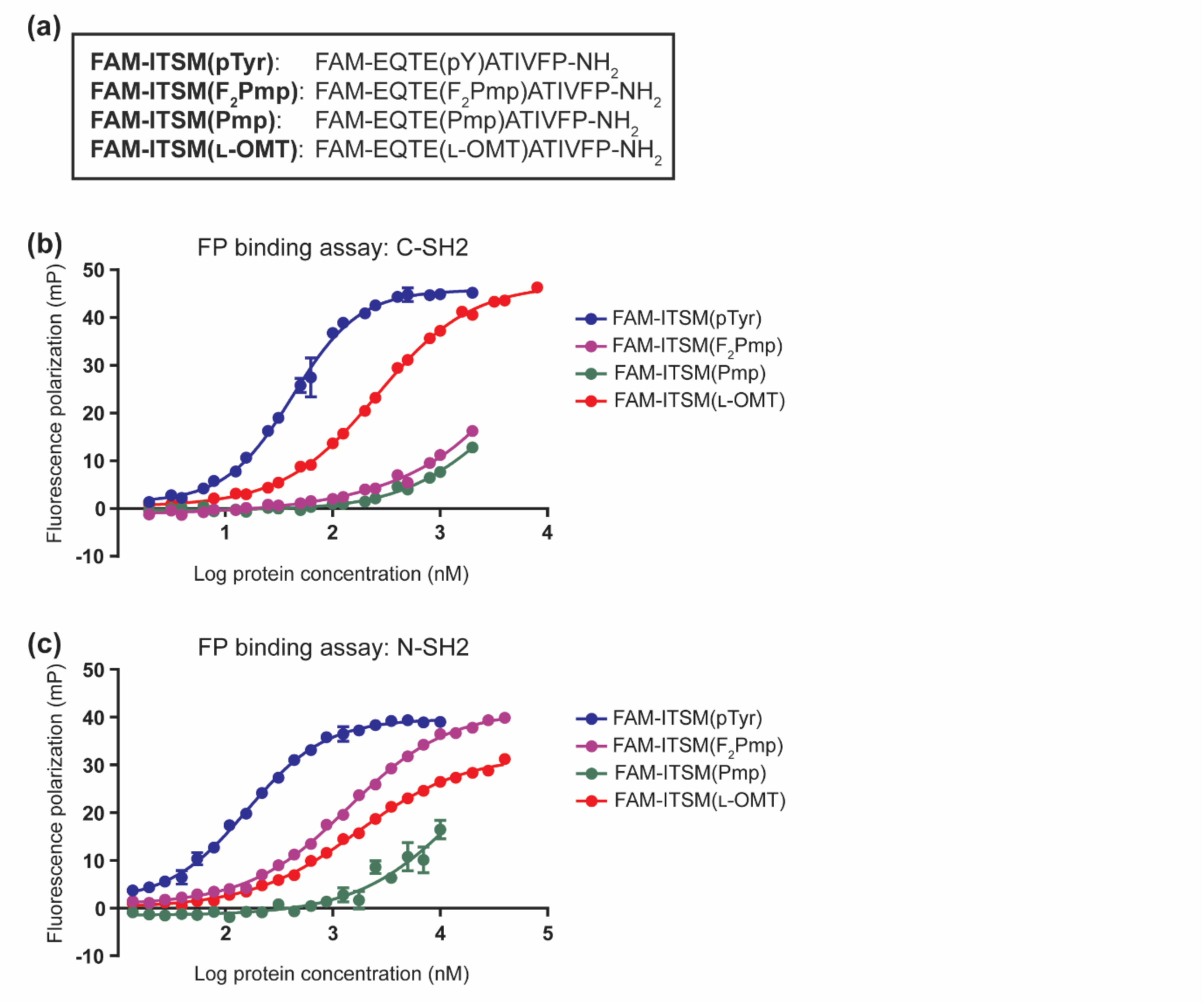

Figure S3: Incorporation of pTyr mimetics into the ITSM peptide sequence.
(a) Amino acid sequence of 5(6)-carboxyfluorescein (FAM)-labelled ITSM peptides containing pTyr or non-hydrolysable pTyr mimetics: phosphonomethyl phenylalanine (Pmp), phosphonodifluoromethyl phenylalanine (F_2Pmp) or L-O-malonyltyrosine (L-OMT). **(b, c)** Fluorescence polarization (FP) assay measuring the binding affinity between increasing concentrations of the SHP2 C-SH2 domain **(a)** or N-SH2 domain **(b)** and 100 nM FAM-ITSM containing pTyr (blue), F_2Pmp (purple), Pmp (green) or L-OMT (red). Data represent the mean of three independent experiments, each performed in technical triplicates, and errors bars represent SEM.

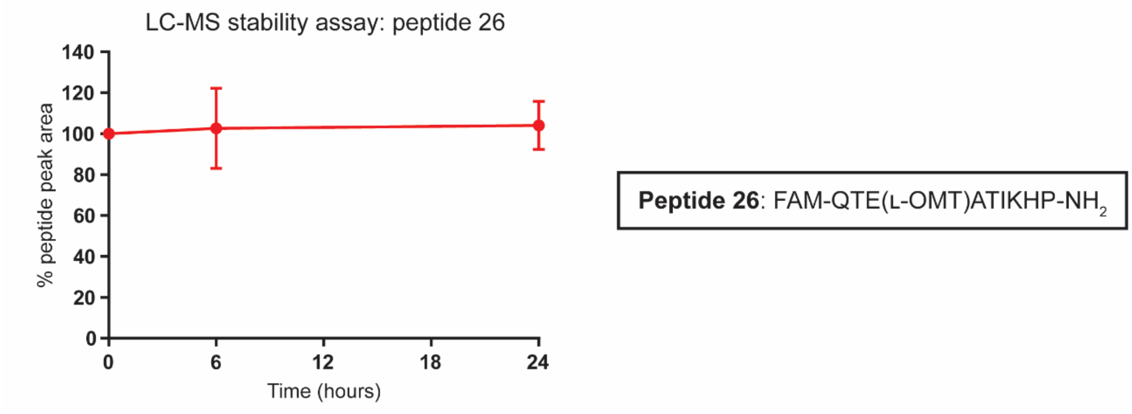

Figure S4: Stability of the C-SH2 inhibitor peptide in Jurkat T cell lysate.

Peptide 26 (200 μM), and FAM (200 μM) as the internal control, were co-incubated with WT Jurkat T cell lysate at 37°C in the dark for 0, 6 or 24 hours then analysed by LC-MS. At each time point, the area under the peak at 254 nm corresponding to peptide 26 was normalised to that of FAM and the ratio at time point zero was set to 100%. Data are from three independent experiments and errors bars represent SEM.

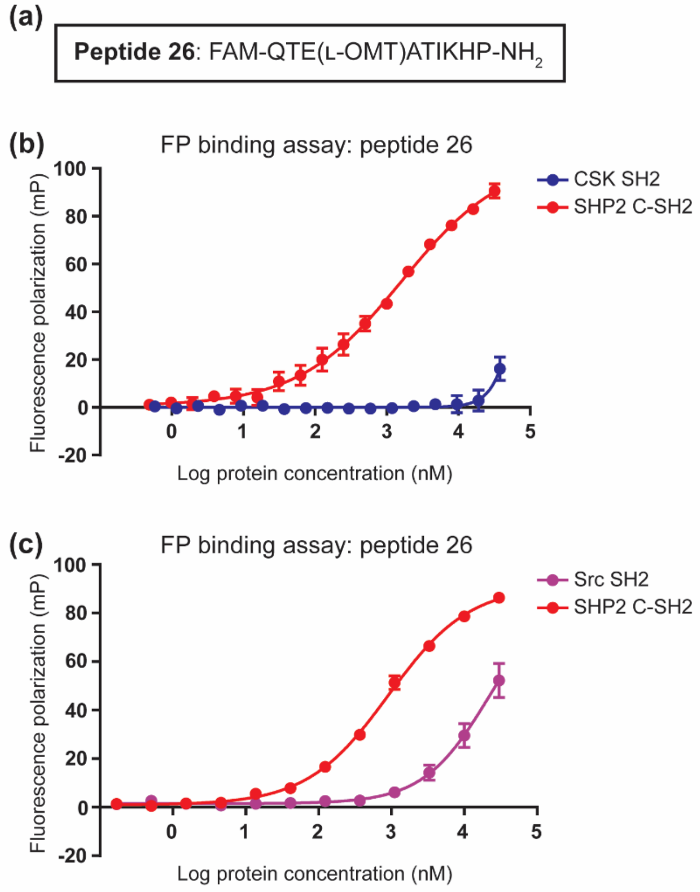

Figure S5: Investigating potential off-targets of the C-SH2 inhibitor peptide.

(a) Amino acid sequence of 5(6)-carboxyfluorescein (FAM)-labelled peptide 26 containing the non-hydrolysable pTyr mimetic L-O-malonyltyrosine (L-OMT). **(b, c)** Fluorescence polarization (FP) assays measuring the binding affinity between 100 nM peptide 26 and increasing concentrations of the CSK SH2 domain (blue) vs SHP2 C-SH2 domain (red) **(b)**, or Src SH2 domain (purple) vs SHP2 C-SH2 domain (red) **(c)**. Data represent the mean of three independent experiments, each performed in technical triplicates, and errors bars represent SEM.

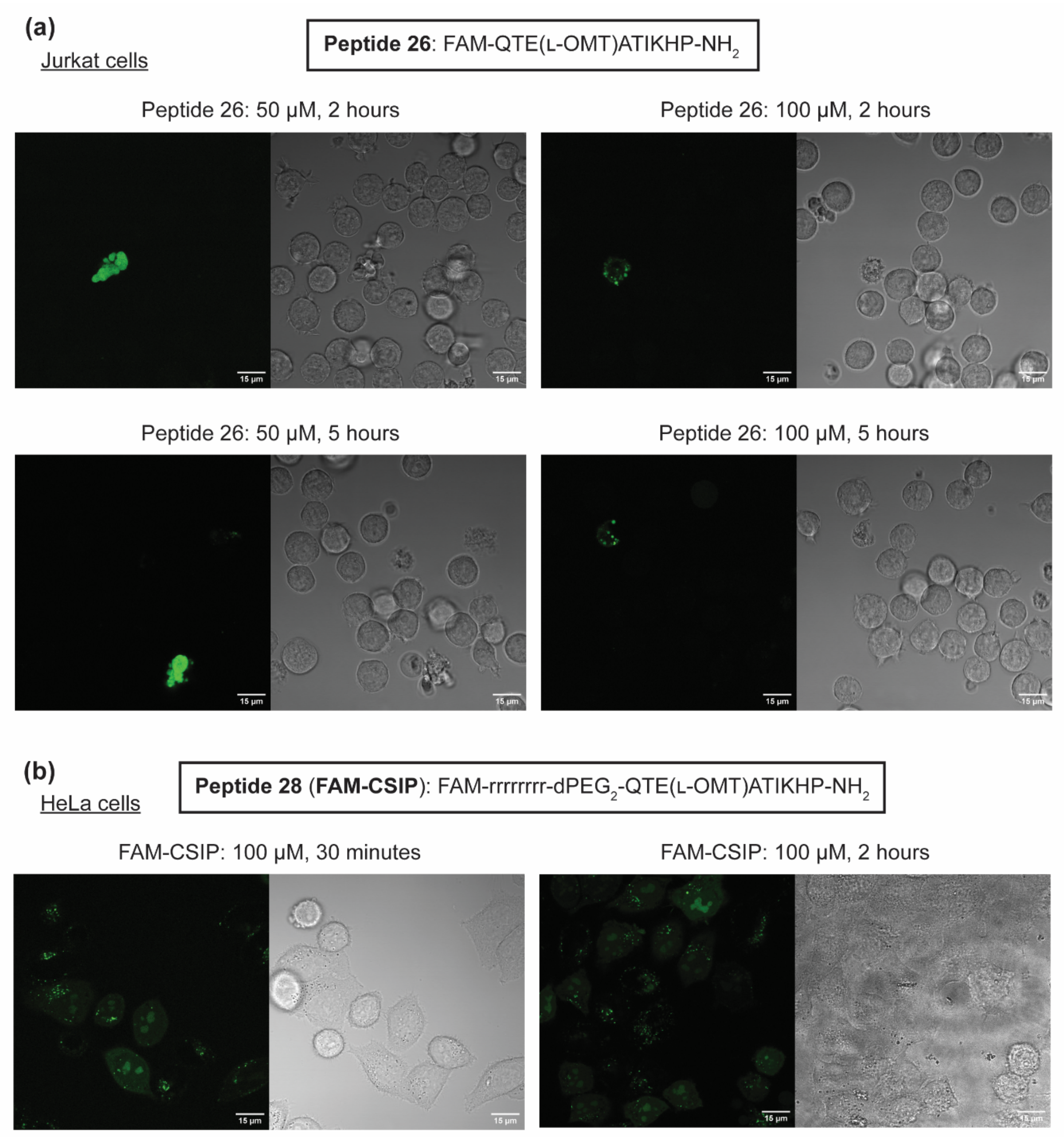

Figure S6: Cell permeability of the C-SH2 inhibitor peptide in Jurkat and HeLa cells.
WT Jurkat T cells were incubated with peptide 26 **(a)** or WT HeLa cells were incubated with FAM-CSIP (peptide 28) **(b)** at the indicated concentrations and for the indicated time points. Cells were imaged by confocal fluorescence microscopy to visualise the cellular uptake of the peptide via its FAM group.

Fluorescence (left) and transmission (right) images are shown and scale bars are 15 µm. Representative images are shown from three or four independent experiments.

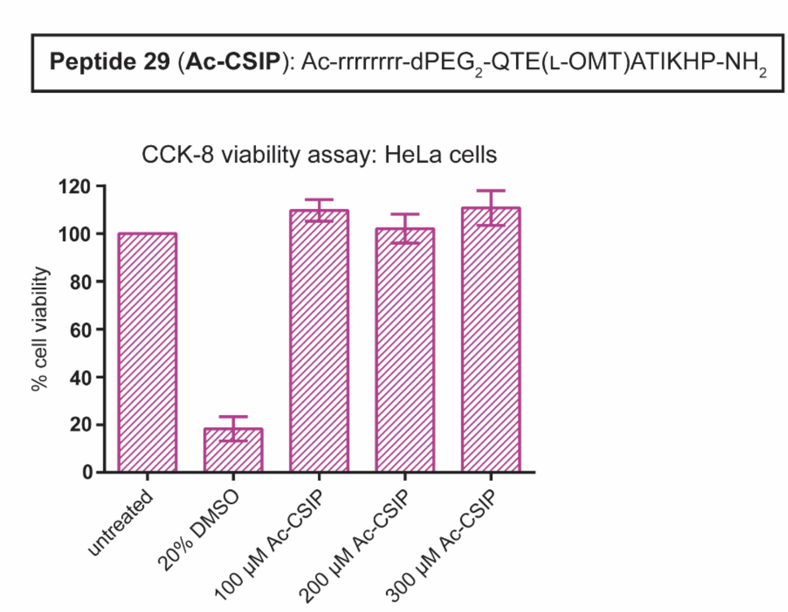

Figure S7: Cytotoxicity of the C-SH2 inhibitor peptide in HeLa cells.

Cell Counting Kit-8 (CCK-8) assay evaluating the cytotoxicity of 100 µM to 300 µM Ac-CSIP (peptide 29) following 16 hour incubation with WT HeLa cells. Vehicle (H_2O) treated cells were used as the negative control and set to 100% viability, while cells treated with 20% DMSO to induce cell death were used as the positive control. Data represent the mean of three or four independent experiments, each performed in technical triplicates, and errors bars represent SEM.

Experimental Section

List of Abbreviations

Ac_2O	acetic anhydride
ACN	acetonitrile
DCM	dichloromethane
DIPEA	*N, N*-diisopropylethylamine
DMF	dimethylformamide
dPEG	discrete polyethylene glycol
ESI	electrospray ionisation
Et_2O	diethyl ether
F_2Pmp	phosphono(difluoromethyl)phenylalanine
FAM	5/6-carboxyfluorescein
Fmoc	fluorenylmethoxycarbonyl
H_2O	water
HBTU	2-(1*H*-benzotriazol-1-yl)-1,1,3,3-tetramethyluronium hexafluorophosphate
HOBt	hydroxybenzotriazole
HPLC	high-performance liquid chromatography
LC-MS	liquid chromatography-mass spectrometry
L-OMT	L-*O*-malonyltyrosine
MS	mass spectrometry
NMM	*N*-methylmorpholine
Pmp	phosphonomethylphenylalanine
SPPS	solid-phase peptide synthesis
TFA	trifluoroacetic acid
TIPS	triisopropyl silane

Chemical Synthesis and Analytical Data of the Peptides

1. Materials

All natural amino acids and rink amide resin were purchased from Novabiochem. The unnatural amino acids were purchased from the following suppliers: Pmp from Iris Biotech, L-OMT from Bachem and the starting materials for F_2Pmp from Sigma or abcr, which was then synthesised following a previously published procedure.[2] All other reagents were purchased from Sigma or Roth.

2. Peptide synthesis

2.1. Automated peptide synthesis

Automated peptide synthesis was performed on a MultiPep RSi pipetting robot (Intavis Bioanalytical Instruments) using Fmoc-protected amino acids and rink amide resin, following the principles of solid-phase peptide synthesis (SPPS) from *C*- to *N*-terminus using the following protocol:

Amino acids were coupled twice for 30 min using Fmoc-amino acids (4 eq.), HBTU (4 eq.), HOBt (0.2 M) and NMM (4 eq.) in DMF, except following the coupling of any unnatural amino acids where the coupling time was prolonged to 90 min. The capping step was conducted using a capping mixture (5% v/v Ac_2O, 5% v/v lutidine in DMF). Fmoc deprotection was performed using 20% v/v piperidine in DMF for 3 min and then 20% v/v piperidine in DMF for 14 min. Peptides were deprotected and cleaved from the resin by gently shaking in a cleavage cocktail (95% TFA, 2.5% TIPS, 2.5% H_2O) overnight. Peptides were precipitated in cold Et_2O and centrifuged (Heraeus Megafuge 11R Centrifuge, 4000 rpm, 5 min, 4°C). Peptides were then analysed by LC-MS and purified by preparative HPLC.

The scale of each synthesis differed based on the sequence, in a manner of 25 µmol for unnatural amino acid incorporated peptides and 50 µmol for peptides with only natural amino acids. The incorporation of unnatural amino acids, FAM, $dPEG_2$ linker and cysteine into the respective peptides were performed manually, as described below.

2.2. Manual peptide synthesis for unnatural amino acid and $dPEG_2$ linker incorporation

For the coupling of unnatural amino acids or $dPEG_2$ linker, 2 mL of DMF was added to the dry resin containing the protected side chain peptide with the open *N*-terminus and was shaken at room temperature for at least 5 min. A reaction mixture containing the desired compound to be coupled (3 eq.), HBTU (3 eq.), HOBT (3 eq.) and NMM (6 eq.) were dissolved in 2 mL of DMF for 10 min. After removing the DMF from the syringe, the reaction mixture was added. Unnatural amino acids and $dPEG_2$ linker were coupled only once overnight due to the high costs and sufficient yield. Following the removal of the solution phase, the solid phase was washed three times with 2 mL of DMF and three times with 2 mL of DCM, each for 5 min on the shaker. Capping was performed twice by adding 2 mL of capping mixture (5% v/v Ac_2O, 5% v/v lutidine in DMF) and incubating for 20 min each time. In between the two capping steps, the solid phase was briefly washed three times with 2 mL of DMF. After capping was completed, the solid phase was washed three times with 2 mL of DMF and three times with 2 mL of DCM. Fmoc deprotection was carried out using the synthesizer as the first step of continuous sequence synthesis.

2.3. Manual peptide synthesis for FAM and cysteine incorporation

The *N*-terminal labelling of peptides with FAM or cysteine was based on the same principle as the manual coupling of other compounds. For FAM-labelled peptides, the coupling steps, as well as all subsequent steps, were performed under the exclusion of light. FAM was coupled using FAM (5 eq.), HOBT (5 eq.), HBTU (5 eq.) and DIPEA (10 eq.) dissolved in 2 mL of DMF. Cysteine was coupled in the same manner using 3 eq. of reagents except DIPEA (6 eq.). FAM and cysteine were coupled twice, whereby the first coupling was performed over three hours and the second coupling overnight. In between the coupling steps, the solid phase was briefly washed three times with 2 mL of DMF. For the cysteine coupling, Fmoc deprotection was performed by adding 1 mL of 20% v/v piperidine in DMF to the resin for 3 min. The solid phase was briefly washed with 2 mL of DMF then 1 mL of 20% v/v piperidine in DMF was added for 10 min. After deprotection was completed, the solid phase was washed three times with 2 mL of DMF and three times with 2 mL of DCM.

3. Analysis and purification of peptides

3.1. Analytical HPLC-MS

Analytical LC-MS measurements were performed on an Agilent Technologies 1260 Infinity I/II HPLC System with a G7117C Diode Array Detector and MS 6120 Single Quadrupole with electrospray ionisation (ESI) source. Separation was performed on a Macherey-Nagel EC 250/4 Nucleodur 100-5 C18 ec (250 x 4.6 mm, 5.0 µm) reversed phase column with a mobile phase of ACN (B) and H_2O (A) with 0.05% TFA as an additive and a flow rate of 0.8 mL/min. Signals were detected at 230 nm. The following standard method was used: 0.0–1.0 min constant at 10% B in A, 1.0–15.0 min linear increase to 90% B, 15.0–18.0 min constant at 90% B, 18.0–18.2 min linear decrease to 10% B, 18.2–20.0 min constant at 10% B.

3.2. Preparative HPLC

The peptides were purified by preparative HPLC on an Agilent Technologies 1260 Infinity I/II HPLC System with a G7165A Multiple Wavelength Detector. Purification was performed on a Macherey-Nagel 125/21 Nucleodur 100-5 C18 ec (125 x 21 mm, 5.0 µm) reversed phase column with a mobile phase of 95% ACN in H_2O (B) and H_2O (A) with 0.05% TFA as an additive. Signals were detected at 230 nm.

4. Peptide characterisation

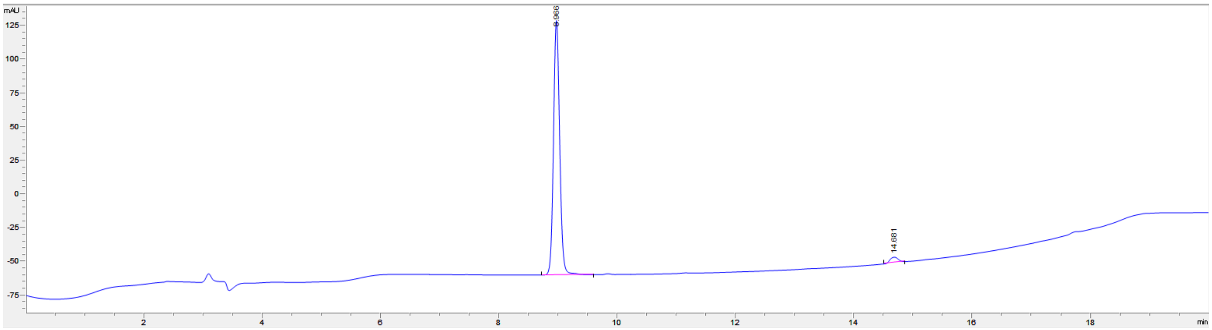

#	Time	Type	Area	Height	Width	Area%	Symmetry
1	8.966	BB	1367.9	188.8	0.1105	97.176	0.852
2	14.681	BB	39.7	4.1	0.1579	2.824	1.158

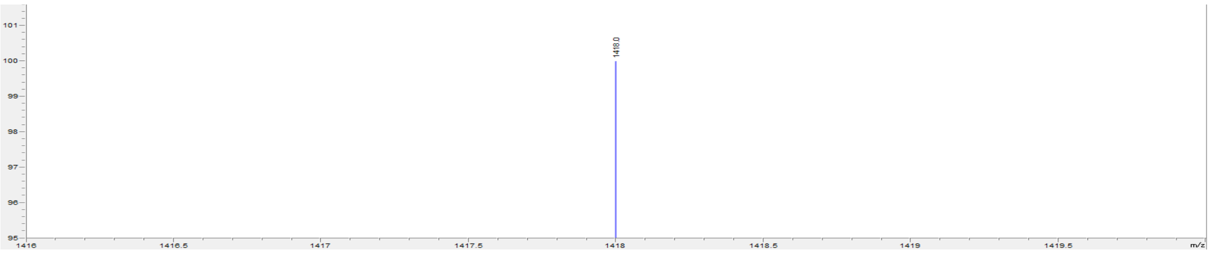

Figure S8: LC-MS analysis of Ac-ITSM(pTyr).

Sequence: Ac-EQTE(pY)ATIVFP-NH_2, calculated mass $[M+H]^+$ = 1419.6, found mass $[M+H]^+$ = 1418.0, retention time: 8.97 min, purity: 97.2%.

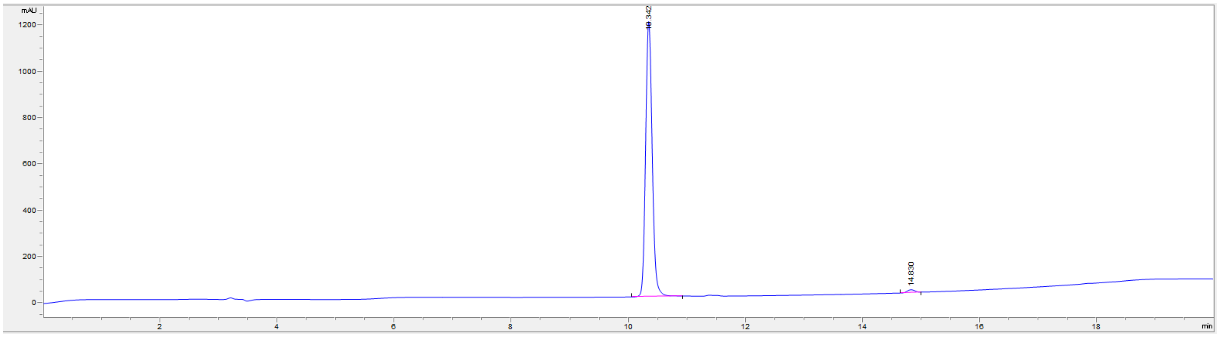

#	Time	Type	Area	Height	Width	Area%	Symmetry
1	10.342	BB	9321.5	1191.4	0.1213	98.837	0.844
2	14.83	BB	109.7	12	0.1486	1.163	1.057

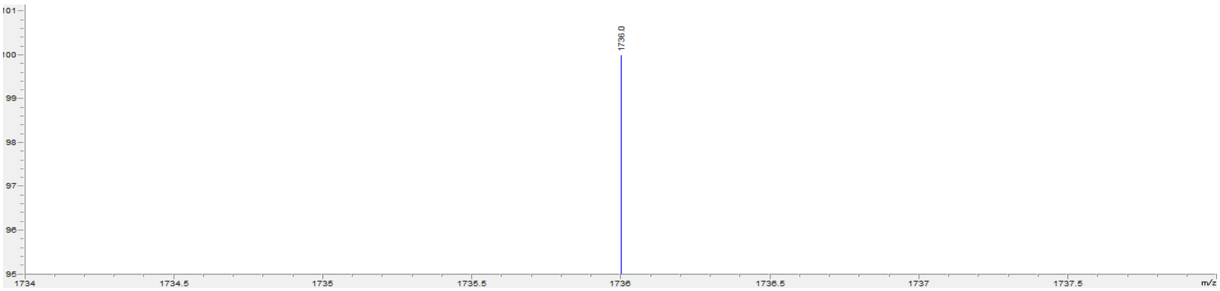

Figure S9: LC-MS analysis of FAM-ITSM(pTyr).

Sequence: FAM-EQTE(pY)ATIVFP-NH_2, calculated mass $[M+H]^+$ = 1735.9, found mass $[M+H]^+$ = 1736.0, retention time: 10.34 min, purity: 98.8%.

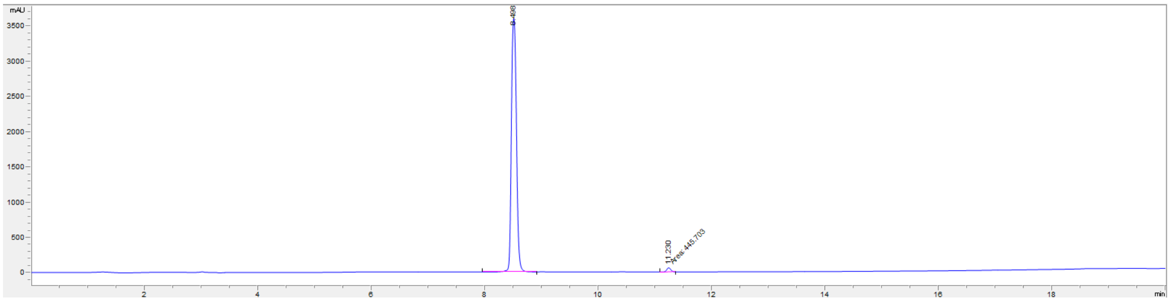

#	Time	Type	Area	Height	Width	Area%	Symmetry
1	8.498	BB	22211.2	3592.4	0.0981	98.033	0.833
2	11.23	MM	445.7	67.6	0.1098	1.967	1.089

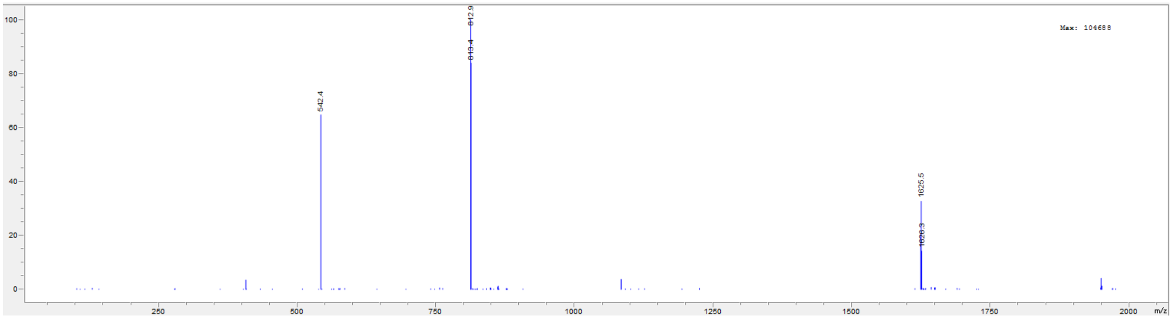

Figure S10: LC-MS analysis of peptide 18.

Sequence: FAM-QTE(pY)ATIKHP-NH_2, calculated mass $[M+H]^+$ = 1625.9, found mass $[M+H]^+$ = 1625.5 $[M+2H]^{2+}$ = 812.9, retention time: 8.50 min, purity: 98.0%.

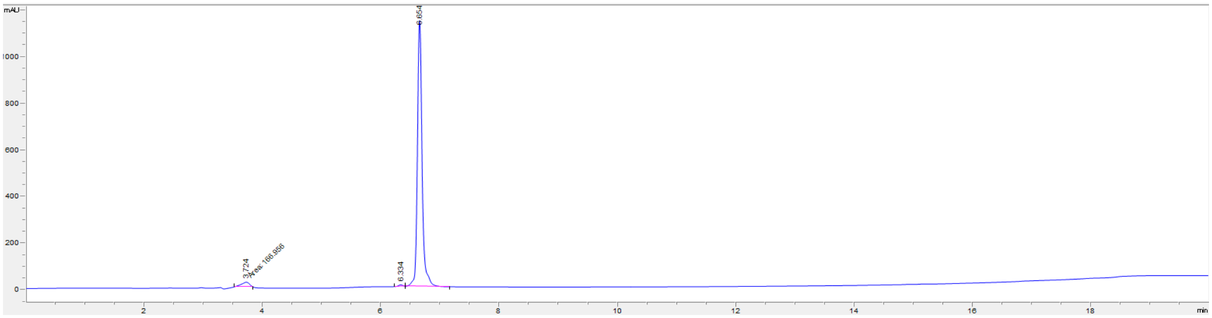

#	Time	Type	Area	Height	Width	Area%	Symmetry
1	3.724	MM	167	18.7	0.1492	2.582	1.61
2	6.334	BB	32.1	7	0.0714	0.497	1.086
3	6.654	BB	6267	1143.7	0.0819	96.921	0.828

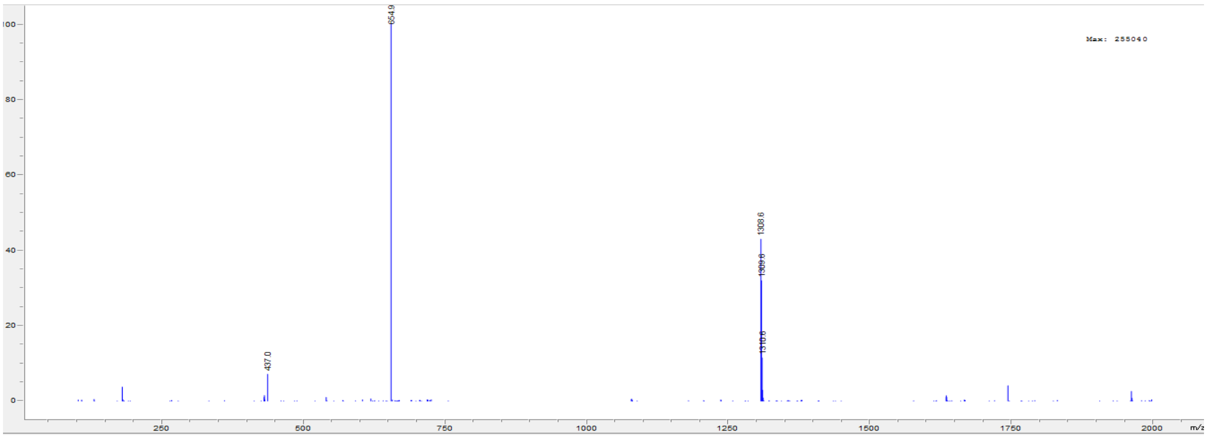

Figure S11: LC-MS analysis of peptide 21.

Sequence: Ac-QTE(pY)ATIKHP-NH_2, calculated mass $[M+H]^+$ = 1308.4, found mass $[M+H]^+$ = 1308.6 $[M+2H]^{2+}$ = 654.9, retention time: 6.65 min, purity: 96.9%.

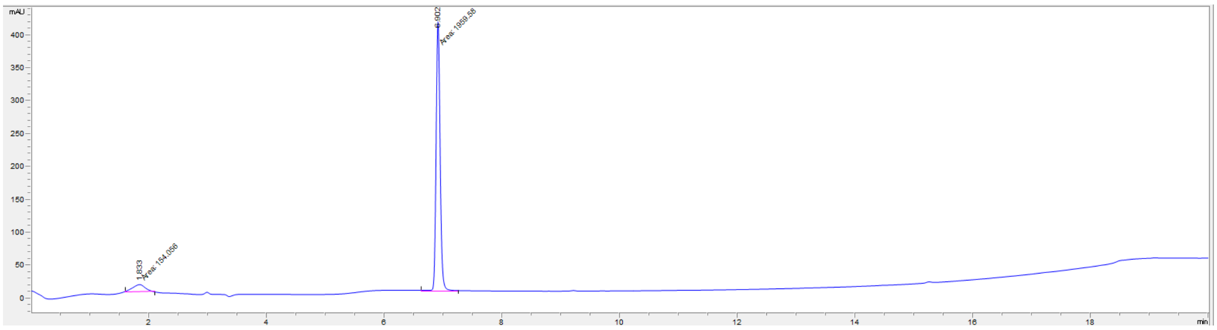

#	Time	Type	Area	Height	Width	Area%	Symmetry
1	1.833	MM	154.1	10.7	0.2396	7.289	1.283
2	6.902	MM	1959.6	410.5	0.0796	92.711	0.873

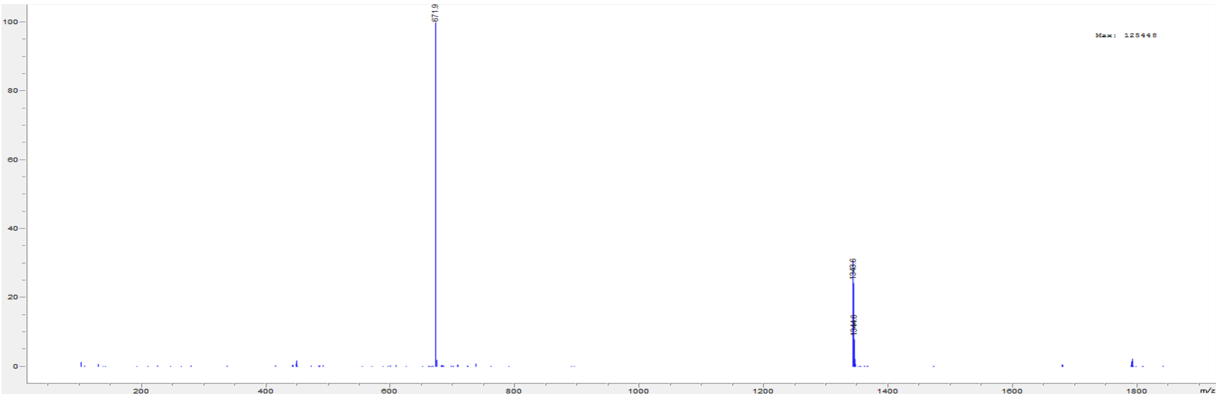

Figure S12: LC-MS analysis of peptide 22.

Sequence: Ac-QTE(F2Pmp)ATIKHP-NH_2, calculated mass $[M+H]^+$ = 1342.6, found mass $[M+H]^+$ = 1343.6 $[M+2H]^{2+}$ = 671.9, retention time: 6.90 min, purity: 92.7%.

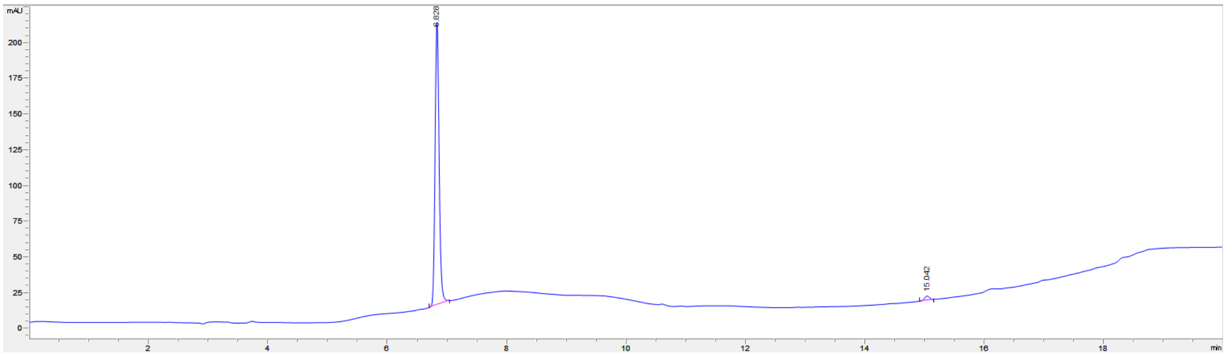

#	Time	Type	Area	Height	Width	Area%	Symmetry
1	6.828	BB	932.3	198.2	0.0729	98.228	0.843
2	15.042	BB	16.8	2.9	0.0906	1.772	0.991

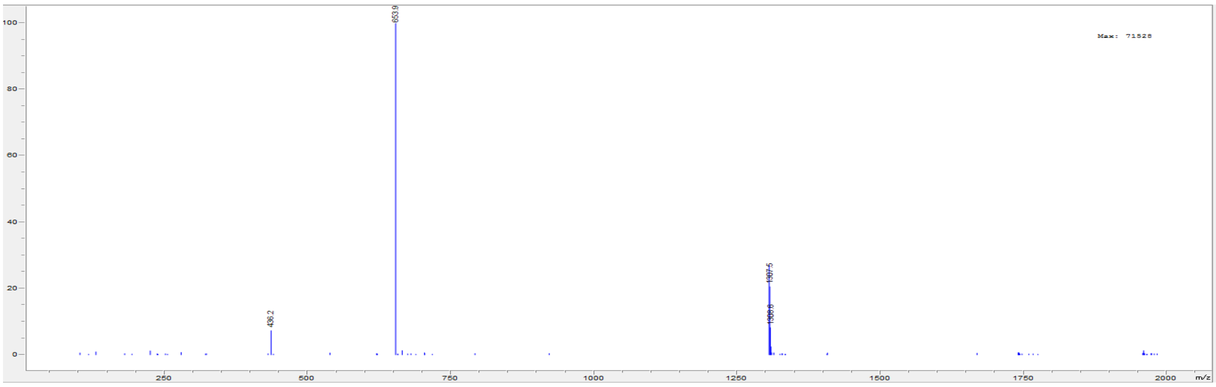

Figure S13: LC-MS analysis of peptide 23.

Sequence: Ac-QTE(Pmp)ATIKHP-NH_2, calculated mass $[M+H]^+$ = 1306.6, found mass $[M+H]^+$ = 1307.5 $[M+2H]^{2+}$ = 653.9, retention time: 6.83 min, purity: 98.2%.

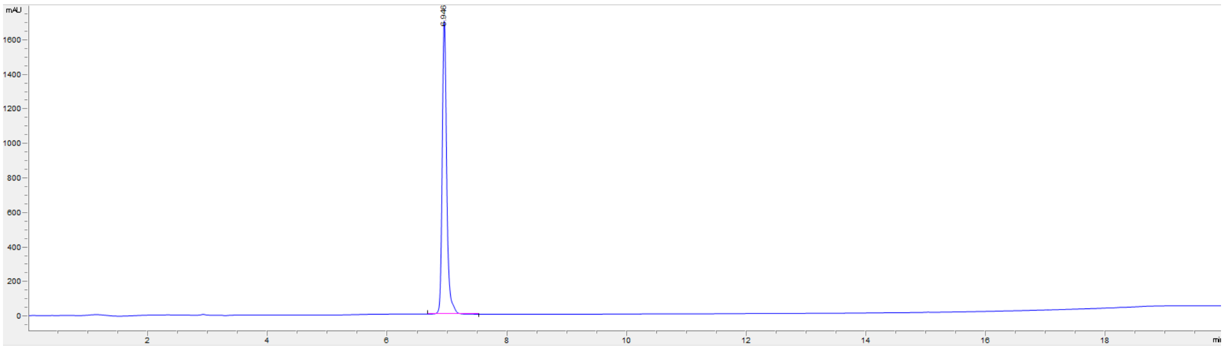

#	Time	Type	Area	Height	Width	Area%	Symmetry
1	6.946	BB	8905.9	1698.7	0.0792	100.000	0.791

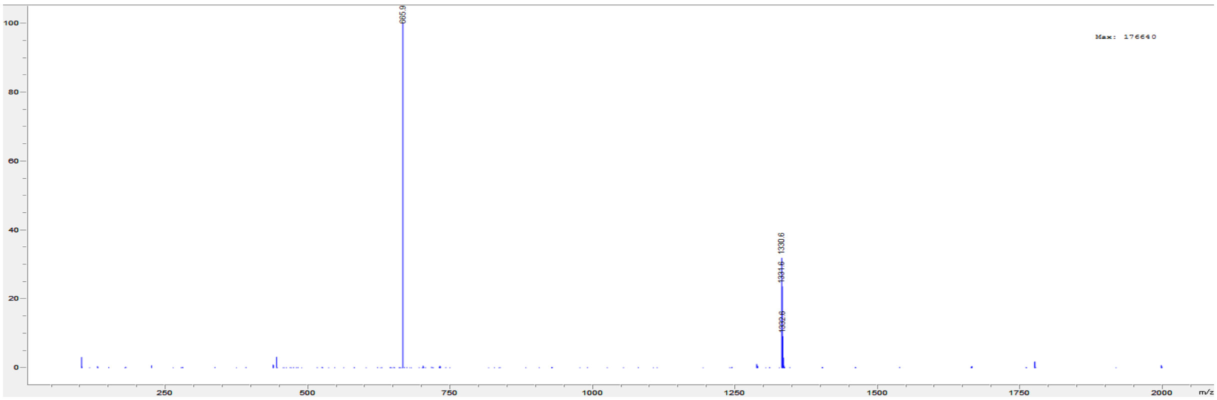

Figure S14: LC-MS analysis of peptide 24.

Sequence: Ac-QTE(L-OMT)ATIKHP-NH_2, calculated mass $[M+H]^+$ = 1331.6, found mass $[M+H]^+$ = 1331.6 $[M+2H]^{2+}$ = 665.9, retention time: 6.95 min, purity: 100%.

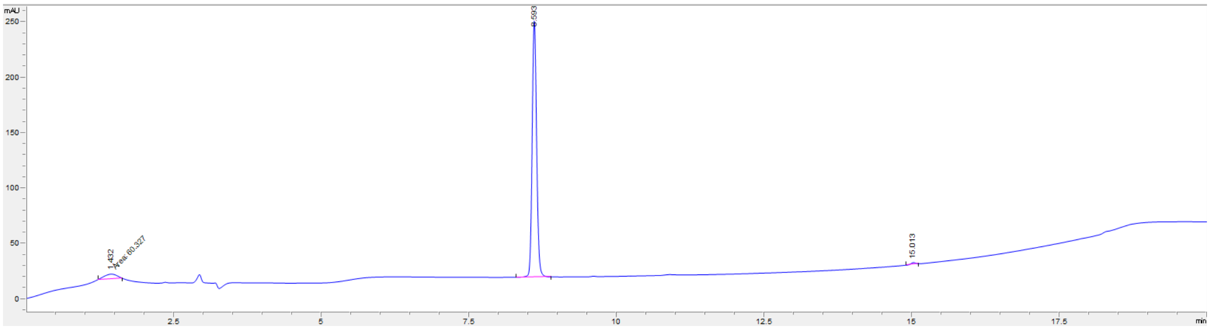

#	Time	Type	Area	Height	Width	Area%	Symmetry
1	1.432	MM	60.3	4.2	0.2411	4.694	1.36
2	8.593	BB	1216.1	231.9	0.0812	94.623	0.874
3	15.013	BB	8.8	1.6	0.0882	0.683	1.111

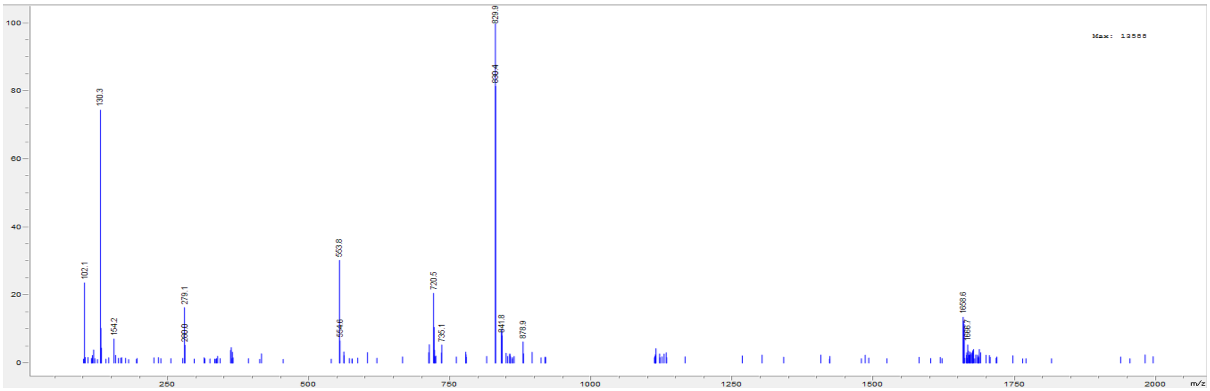

Figure S15: LC-MS analysis of peptide 25.

Sequence: FAM-QTE(F2Pmp)ATIKHP-NH_2, calculated mass $[M+H]^+$ = 1659.9, found mass $[M+H]^+$ = 1658.6 $[M+2H]^{2+}$ = 829.9, retention time: 8.59 min, purity: 94.6%.

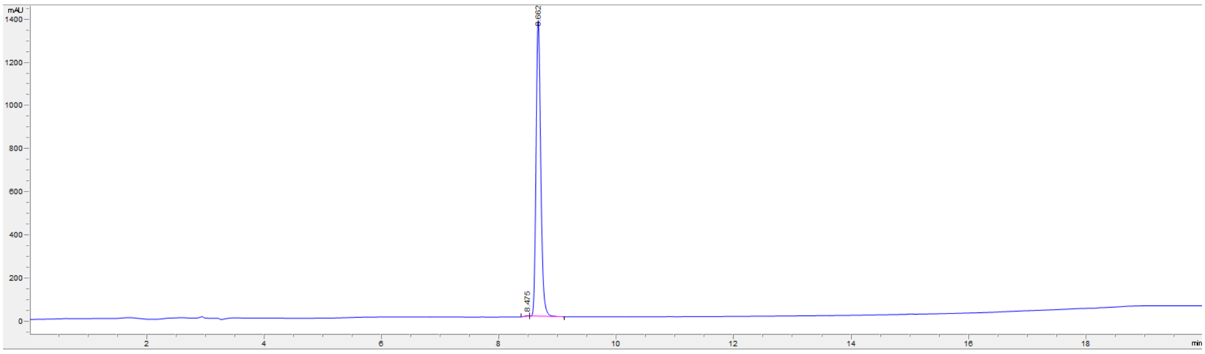

#	Time	Type	Area	Height	Width	Area%	Symmetry
1	8.475	BB	12.4	2.7	0.0728	0.162	1.896
2	8.662	BB	7605.6	1372.1	0.0847	99.838	0.791

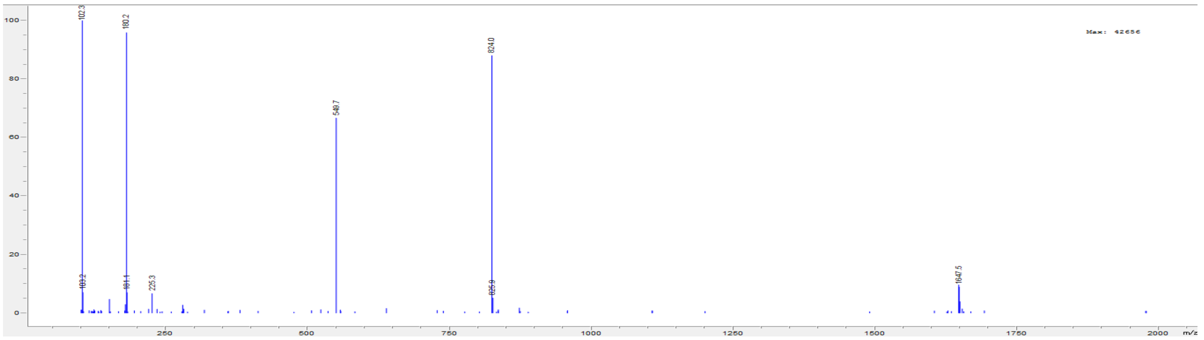

Figure S16: LC-MS analysis of peptide 26.

Sequence: FAM-QTE(L-OMT)ATIKHP-NH_2, calculated mass $[M+H]^+$ = 1647.9, found mass $[M+H]^+$ =1647.5 $[M+2H]^{2+}$ = 824.0, retention time: 8.66 min, purity: 99.8%.

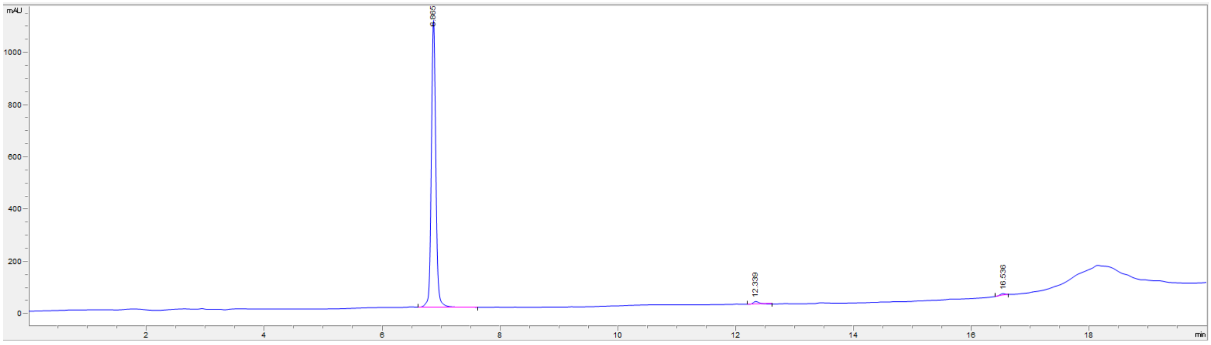

#	Time	Type	Area	Height	Width	Area%	Symmetry
1	6.865	BB	5774.9	1097.2	0.0814	98.077	0.912
2	12.339	BB	74.6	10.1	0.1103	1.267	0.611
3	16.536	BB	38.7	6.1	0.1025	0.657	1.448

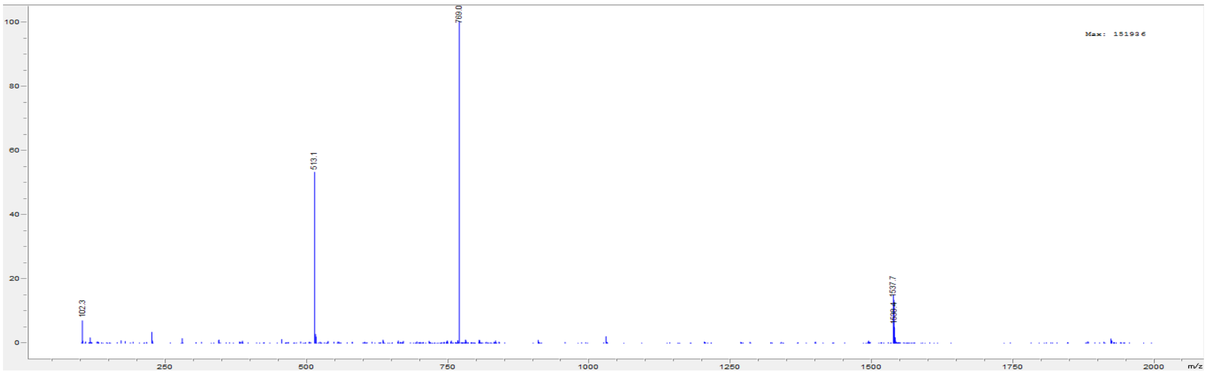

Figure S17: LC-MS analysis of peptide 27.

Sequence: Cys-dPEG$_2$-QTE(L-OMT)ATIKHP-NH$_2$, calculated mass $[M+H]^+$ = 1537.6, found mass $[M+H]^+$ = 1537.7 $[M+2H]^{2+}$ = 769.0, retention time: 6.87 min, purity: 98.1%.

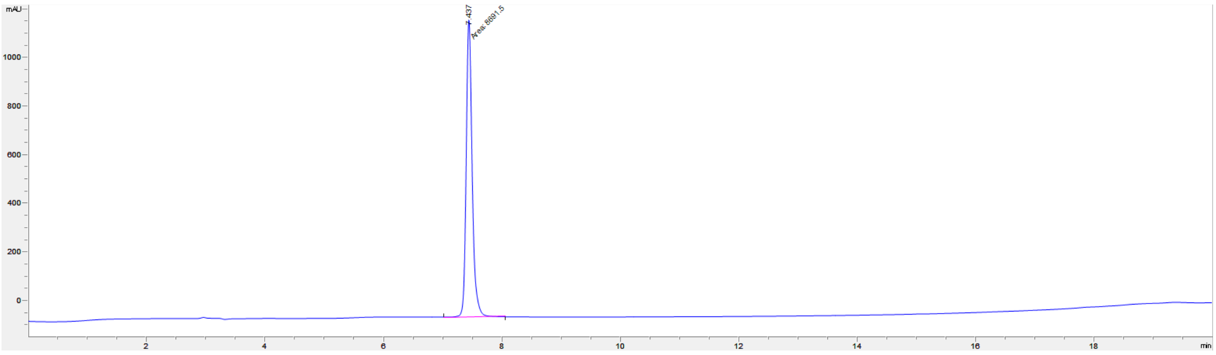

#	Time	Type	Area	Height	Width	Area%	Symmetry
1	7.437	MM	8691.5	1225.8	0.1182	100.000	0.807

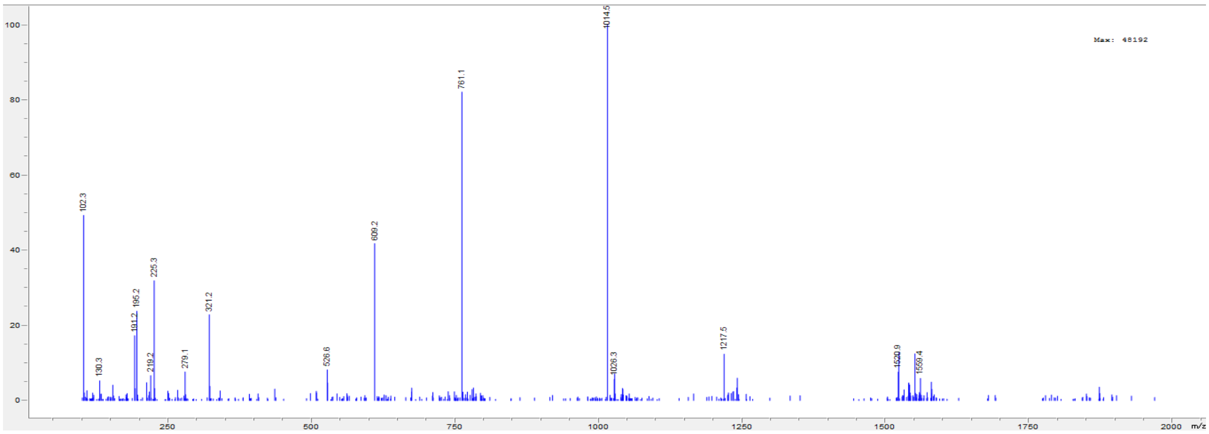

Figure S18: LC-MS analysis of peptide 28.

Sequence: FAM-rrrrrrrr-dPEG$_2$-QTE(L-OMT)ATIKHP-NH$_2$, calculated mass $[M+H]^+$ = 3040.7, found mass $[M+2H]^{2+}$ = 1520.9 $[M+3H]^{3+}$ = 1014.5 $[M+4H]^{4+}$ = 761.1, retention time: 7.44 min, purity: 100%.

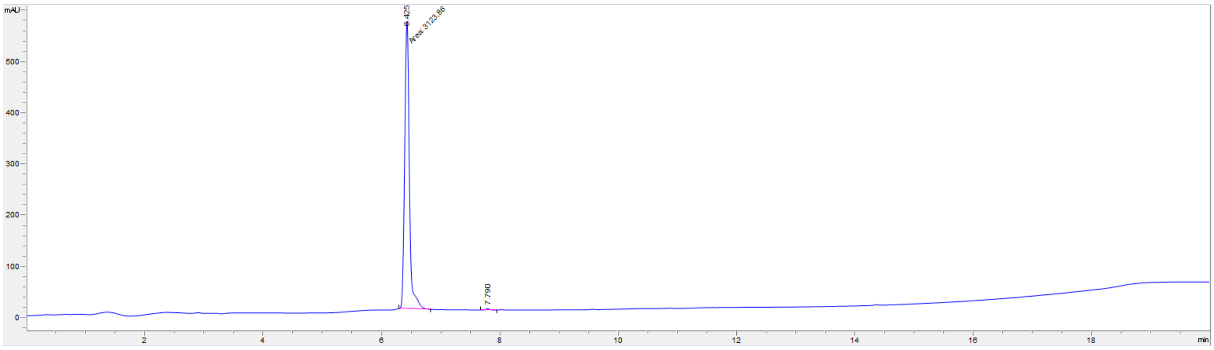

#	Time	Type	Area	Height	Width	Area%	Symmetry
1	6.425	MM	3123.9	566.3	0.0919	99.638	0.894
2	7.79	BB	11.3	2.5	0.07	0.362	0.899

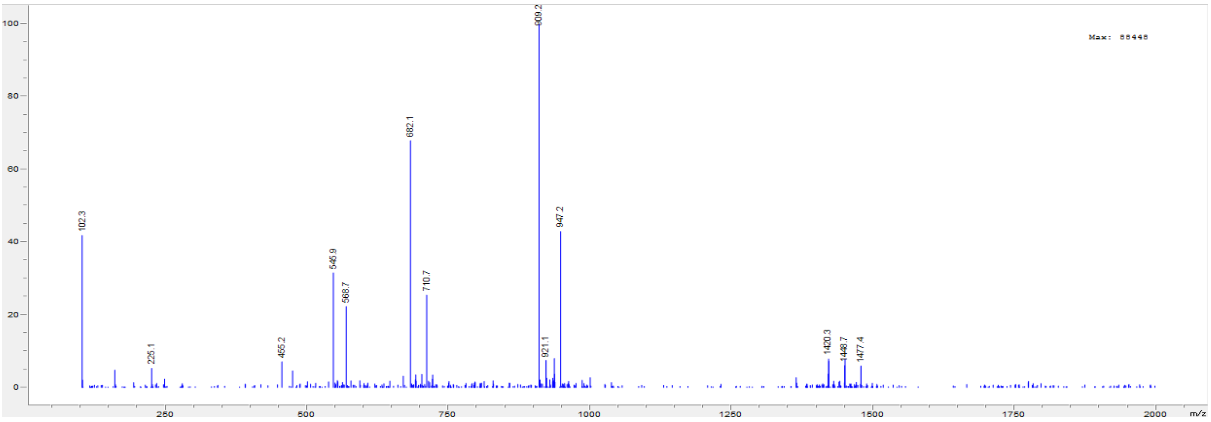

Figure S19: LC-MS analysis of peptide 29.

Sequence: Ac-rrrrrrrr-dPEG$_2$-QTE(L-OMT)ATIKHP-NH$_2$, calculated mass $[M+H]^+$ = 2723.7, found mass $[M+3H]^{3+}$ = 909.2 $[M+4H]^{4+}$ = 682.1, retention time: 6.43 min, purity: 99.6%.

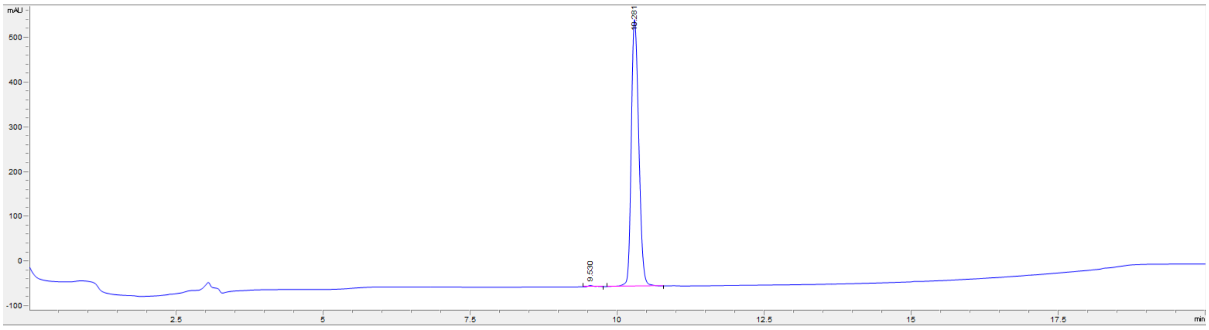

#	Time	Type	Area	Height	Width	Area%	Symmetry
1	9.53	BB	19.6	3	0.0943	0.372	0.598
2	10.281	BB	5256.5	594.9	0.1411	99.628	0.731

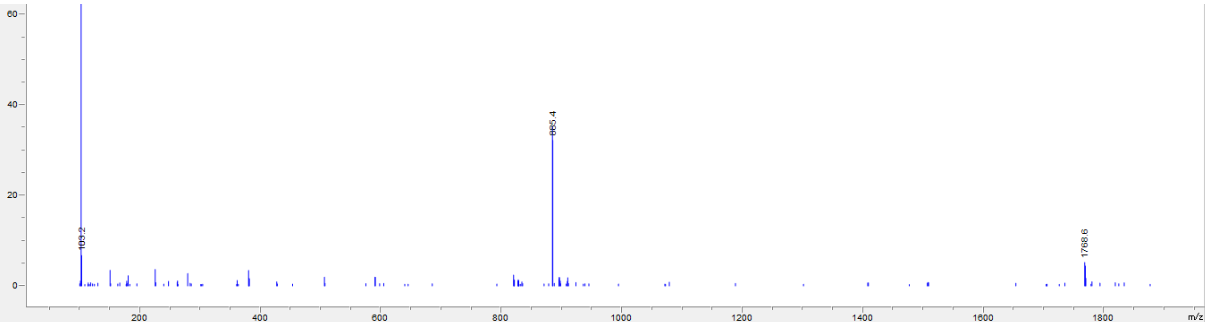

Figure S20: LC-MS analysis of FAM-ITSM(F_2Pmp).

Sequence: FAM-EQTE(F_2Pmp)ATIVFP-NH_2, calculated mass $[M+H]^+$ = 1767.7, found mass $[M+H]^+$ = 1768.6 $[M+2H]^{2+}$ = 885.4, retention time: 10.28 min, purity: 99.6%.

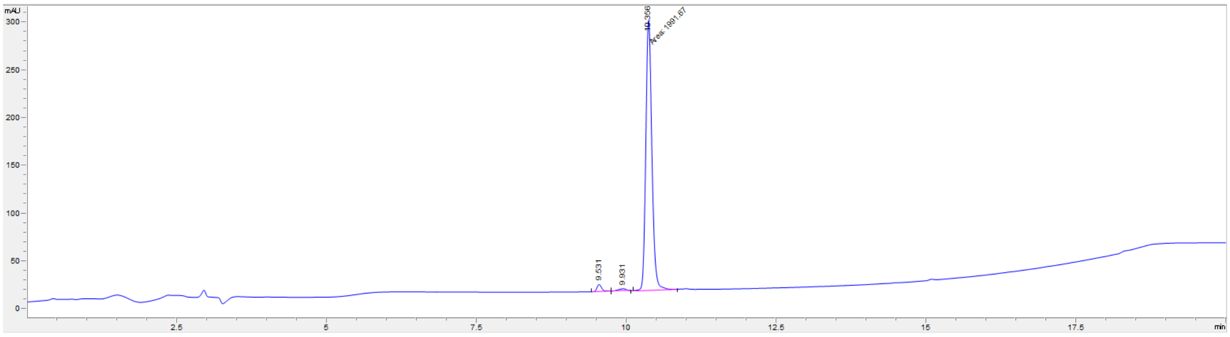

#	Time	Type	Area	Height	Width	Area%	Symmetry
1	9.531	BB	39.5	7.3	0.0828	1.927	0.79
2	9.931	BB	19.4	2.2	0.1266	0.948	1.472
3	10.356	MM	1991.7	282.9	0.1173	97.124	0.819

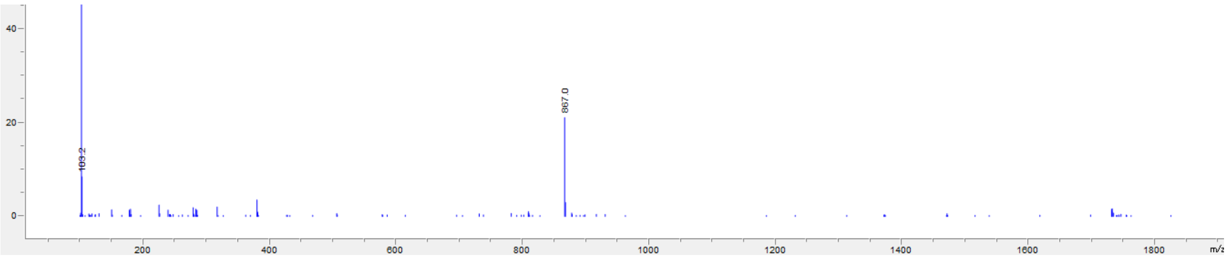

Figure S21: LC-MS analysis of FAM-ITSM(Pmp).

Sequence: FAM-EQTE(Pmp)ATIVFP-NH_2, calculated mass $[M+H]^+$ = 1732.7, found mass $[M+2H]^{2+}$ = 867.0, retention time: 10.36 min, purity: 97.1%.

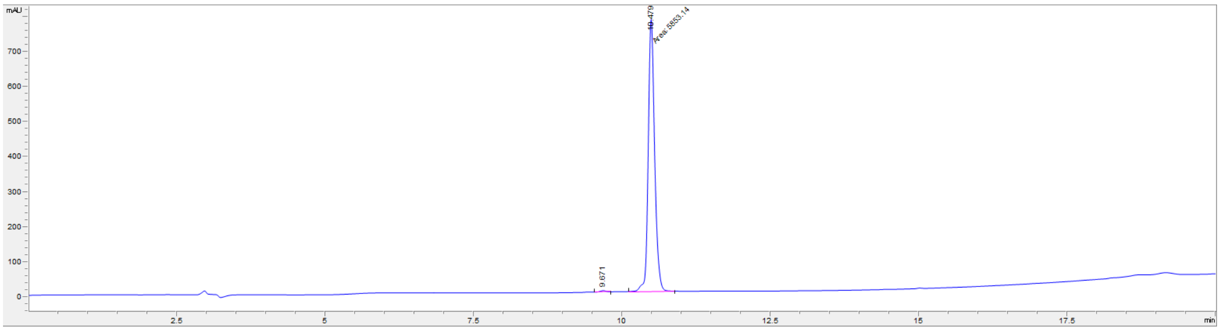

#	Time	Type	Area	Height	Width	Area%	Symmetry
1	9.671	BB	24.4	3.8	0.0998	0.415	0.982
2	10.479	MM	5853.1	776.6	0.1256	99.585	0.82

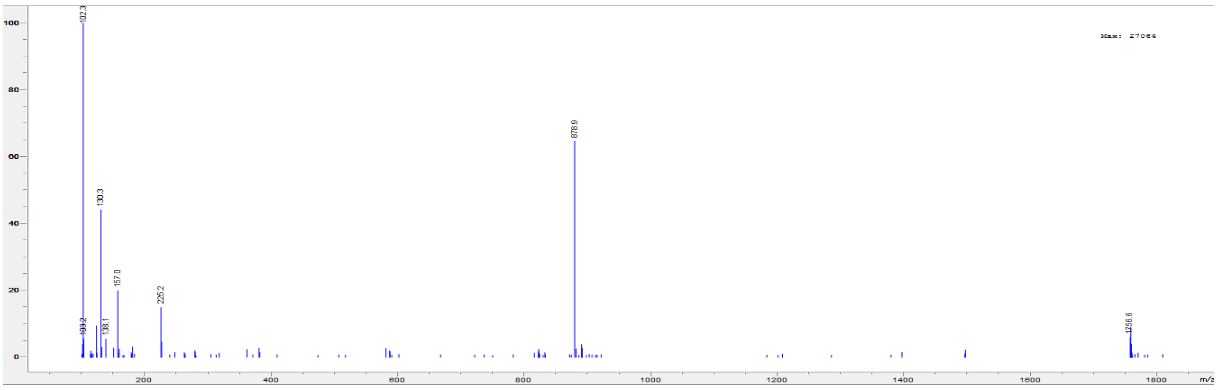

Figure S22: LC-MS analysis of FAM-ITSM(L-OMT).

Sequence: FAM-EQTE(L-OMT)ATIVFP-NH$_2$, calculated mass [M+H]$^+$ = 1756.7, found mass [M+H]$^+$ = 1756.6 [M+2H]$^{2+}$ = 878.9, retention time: 10.48 min, purity: 99.6%.

Biochemical Experiments

1. SHP2 protein preparation

SHP2 N-SH2 domain (1–105), C-SH2 domain (106–220), PTP domain (224–541) and full-length (without the *C*-terminal tail; 1–525) proteins were prepared as previously described.[1] Briefly, the N-SH2 domain and C-SH2 domain were cloned into the pETM22 vector and expressed in BL21(DE3) *E. coli* cells. The PTP domain was cloned into the pETM22 vector and expressed in BL21(DE3) Tuner *E. coli* cells. Full-length (without the *C*-terminal tail) protein was cloned into the pETM11-SUMO3 vector and expressed in BL21(DE3) *E. coli* cells. The N-SH2, C-SH2 and PTP domains were purified by Ni^{2+}-affinity chromatography, followed by 3C protease cleavage, before a second Ni^{2+}-affinity chromatography step, and finally size exclusion chromatography. Full-length (without the *C*-terminal tail) protein was purified by Ni^{2+}-affinity chromatography, followed by SENP2 protease cleavage, before ion-exchange chromatography, and finally size exclusion chromatography.

2. CSK protein preparation

2.1. Cloning

DNA encoding the CSK SH2 domain (82–171) was cloned into the pETM22 vector (EMBL), encoding an *N*-terminal His_6-tag linked with a 3C protease cleavage site to the protein of interest and resistance to kanamycin. To amplify the CSK SH2 domain sequence from the pcFLAG-CSK-WT vector (gift from Lars Rönnstrand; Addgene plasmid #74502),[3] forward and reverse oligonucleotide primers were designed and ordered (Sigma-Aldrich).

Forward Primer: 5‘-CCATGCCATGGCTTGGTTCCACGGCAAG-3’

Reverse Primer: 5’-CCGCTCGAGttatcaCTTTGGTTTAATGAGGCGC-3’

Lyophilised oligonucleotide primers were dissolved in ddH_2O to obtain a concentration of 10 µM. Insert DNA was amplified from template DNA by PCR in a total volume of 50 µL containing 10 ng of template DNA, 2.5 µL of 10 µM forward primer, 2.5 µL of 10 µM reverse primer, 1 µL of 10 mM dNTPs (Thermo Scientific), 0.5 µL of Phusion DNA

Polymerase (Thermo Scientific), 10 µL of 5X Phusion Buffer (Thermo Scientific) and ddH_2O. PCR was performed using the cycling conditions: 98°C for 30 seconds followed by 30 cycles of 98°C for 15 seconds, 59.1°C for 45 seconds and 72°C for 10 seconds then 72°C for 10 minutes. PCR product was purified using the NucleoSpin Gel and PCR Clean-up Kit (Macherey-Nagel) according to the manufacturer's instructions and the concentration measured at 260 nm on a NanoDrop 2000c Spectrophotometer (Thermo Scientific). PCR product and pETM22 vector were separately digested in a total volume of 20 µL containing 100 ng of purified DNA, 1 µL of FastDigest *Nco*I restriction enzyme (Thermo Scientific), 1 µL of FastDigest *Xho*I restriction enzyme (Thermo Scientific), 2 µL of 10X FastDigest Green Buffer (Thermo Scientific) and ddH_2O for 1 hour at 37°C. Digested products were separated by DNA agarose gel electrophoresis using 1% w/v agarose (Roth) in TAE buffer (40 mM Tris, 20 mM acetic acid, 1 mM EDTA) containing 1:10000 SYBR Safe DNA Gel Stain (Invitrogen). Digested insert DNA and pETM22 vector were excised and extracted from the agarose gel using the NucleoSpin Gel and PCR Clean-up Kit (Macherey-Nagel) according to the manufacturer's instructions and their concentration measured at 260 nm on a NanoDrop 2000c Spectrophotometer (Thermo Scientific). Purified insert DNA was ligated into the purified pETM22 vector in a total volume of 20 µL containing 50 ng of vector and 400 ng of insert DNA (ratio 1:8), 1 µL of T4 DNA Ligase (Thermo Scientific), 2 µL of 10X T4 DNA Ligase Buffer (Thermo Scientific) and ddH_2O for 1 hour at room temperature. The ligation reaction mixture was transformed into chemically competent TOP10 *E. coli* cells (prepared in-house; available from Thermo Scientific) via heat shock, by adding 100 ng of ligation to 50 µL of TOP10 cells and incubating for 30 minutes on ice, before the cells were heat shocked for 45 seconds at 42°C and 2 minutes on ice. 250 µL of LB medium was added and incubated shaking at 400 rpm for 1 hour at 37°C. 150 µL of transformed cells were plated onto an LB-agar plate containing 50 µg/mL kanamycin (Roth) and incubated overnight at 37°C. Single colonies were picked and used to inoculate overnight cultures consisting of 6 mL of LB medium containing 50 µg/mL kanamycin (Roth) grown shaking at 200 rpm overnight at 37°C. Plasmid DNA was extracted using the NucleoSpin Plasmid (No Lid) Kit (Macherey-Nagel) according to the manufacturer's instructions and the concentration measured at 260 nm on a NanoDrop 2000c Spectrophotometer (Thermo Scientific). 20 µL of 30–50 ng/µL plasmid were sent to Eurofins Genomics for sequencing.

2.2. Expression

Plasmid DNA was transformed into chemically competent BL21(DE3) *E. coli* cells (prepared in-house; strain from EMBL) via heat shock, as detailed above. Transformed cells were plated onto an LB-agar plate containing 50 µg/mL kanamycin (Roth) and incubated overnight at 37°C. Single colonies were picked and used to inoculate a starter culture consisting of 100 mL of LB medium containing 50 µg/mL kanamycin (Roth) grown shaking at 200 rpm overnight at 37°C. 20 mL of the starter culture was then used to inoculate the expression culture consisting of 1 L of LB medium containing 50 µg/mL kanamycin (Roth) grown shaking at 200 rpm at 37°C. When the OD_{600} reached between 0.6–0.8, protein overexpression was induced with 0.1 mM IPTG (Roth) at 20°C overnight. Protein overexpression before and after IPTG induction was analysed by SDS-PAGE using NuPAGE 4-12% Bis-Tris Protein Gels (Invitrogen) run in MOPS buffer (50 mM MOPS, 50 mM Tris-HCl pH 7.7, 1 mM EDTA, 0.1% SDS) followed by Coomassie staining using Coomassie Brilliant Blue (Roth). Bacterial culture was harvested by centrifugation at 6000 x *g* for 20 minutes at 4°C. Bacterial pellet was transferred to a 50 mL tube, centrifuged at 4000 x *g* for 15 minutes at 4°C to remove residual liquid and stored at -20°C if not used immediately for protein purification.

2.3. Purification

Bacterial cell pellet was resuspended in 20 mL of lysis buffer (50 mM Tris-HCl pH 7.5, 500 mM NaCl, 30 mM imidazole) containing cOmplete EDTA-free Protease Inhibitor Cocktail (Roche), lysed by sonication and cleared by centrifugation at 16000 x *g* for 45 minutes at 4°C. In a 20 mL Econo-Pca Chromatography Column (Bio-Rad), 2 mL Ni-NTA Agarose (Invitrogen) as a 50% slurry in EtOH was loaded. The column was washed with 10 mL H_2O to remove the EtOH and equilibrated with 10 mL binding buffer (50 mM Tris-HCl pH 7.5, 500 mM NaCl, 30 mM imidazole). The lysate supernatant was loaded onto the column and incubated for 1 hour at 4°C on a rocker. The column was washed 3 times with 20 mL binding buffer before the protein was eluted with 5 mL elution buffer 1 (50 mM Tris-HCl pH 7.5, 500 mM NaCl, 200 mM imidazole) and the impurities were eluted with 5 mL elution buffer 2 (50 mM Tris-HCl pH 7.5, 500 mM NaCl, 500 mM imidazole). The column was washed with 5 mL binding

buffer and stored in 5 mL binding buffer at 4°C. His-tagged 3C protease (0.5 mg; EMBL) was added to the pooled eluted fractions and dialysed using Spectra Por Dialysis Tubing (MWCO 6-8 kDa, 23 mm; Spectrum Labs) in 1 L of binding buffer at 4°C for 3 hours, then overnight in fresh buffer. A new column was prepared by loading 2 mL Ni-NTA Agarose (Invitrogen) as a 50% slurry in EtOH. The column was washed with 10 mL H_2O to remove the EtOH and equilibrated with 10 mL binding buffer. The protein/3C protease mixture was loaded onto the column and incubated for 1 hour at 4°C on a rocker. The flow through containing the protein was collected, the column was washed with 100 mL binding buffer and the 3C protease was eluted with 5 mL elution buffer. Protein purification was monitored by SDS-PAGE using NuPAGE 4-12% Bis-Tris Protein Gels (Invitrogen) run in MOPS buffer (50 mM MOPS, 50 mM Tris-HCl pH 7.7, 1 mM EDTA, 0.1% SDS) followed by Coomassie staining using Coomassie Brilliant Blue (Roth). The purified protein was dialysed into the final storage buffer (50 mM HEPES pH 7.5, 150 mM NaCl, 2 mM DTT, 20% glycerol). Aliquots were prepared and stored at -80°C until required.

3. Src protein preparation

Src SH2 domain (142–249) protein was prepared as previously described.[4] Briefly, the Src SH2 domain was cloned into the pGEX-6P-3 vector, expressed in BL21(DE3) *E. coli* cells and purified by glutathione-affinity chromatography. To cleave the GST tag, a modified procedure was performed using His-tagged 3C protease (EMBL), followed by Ni^{2+}-affinity chromatography (Ni-NTA Agarose; Invitrogen) to remove the protease and then glutathione-affinity chromatography (Glutathione Sepharose 4B; GE Healthcare) to remove the GST.

4. Fluorescence polarization

Fluorescence polarization (FP) was used to measure the binding of FAM-labelled peptides to the SHP2 C-SH2, SHP2 N-SH2, SHP2 PTP, CSK SH2 or Src SH2 domains. Each peptide was added to increasing concentrations of SHP2 C-SH2, SHP2 N-SH2, SHP2 PTP, CSK SH2 or Src SH2 protein in FP buffer (50 mM HEPES pH 7.5, 150 mM NaCl, 1 mM EDTA, 0.05% Tween 20) to give a final peptide

concentration of 100 nM in a final volume of 200 µL. For baseline subtraction, each peptide was added to FP buffer, instead of protein, with a final peptide concentration of 100 nM in a final volume of 200 µL. Samples were mixed gently then 3 x 60 µL of each sample was transferred into triplicate wells in a 96-well plate (OptiPlate-96 F Black; PerkinElmer). The FP was immediately measured on a Synergy H1 Microplate Reader (BioTek) with the excitation and emission monitored at 485 nm and 528 nm, respectively, over 30 minutes. FP values were automatically calculated by the Gen5 3.03 (BioTek) software associated with the microplate reader. Data were analysed in Excel 2016 by subtracting baseline values from FP values at 10 minutes. Data were then further analysed in GraphPad Prism 6 by plotting the baseline subtracted FP values against the protein concentration and fitting the data to a variable slope model of non-linear regression to obtain the K_D values. Data are from three independent experiments, each performed in technical triplicates.

5. Isothermal titration calorimetry

Isothermal titration calorimetry (ITC) was used to measure the binding of the Ac-ITSM(pTyr) to the SHP2 C-SH2 or N-SH2 domain. Stock solutions of 10 µM Ac-ITSM(pTyr), 100 µM C-SH2 domain and 100 µM N-SH2 domain were prepared in ITC assay buffer (20 mM Tris-HCl pH 7.6, 150 mM NaCl) and then degassed under vacuum for 5 minutes. ITC measurements were performed on a MicroCal ITC200 (Malvern Panalytical). 250 µL of 100 µM SH2 domain was added to the sample cell, while H_2O was added to the reference cell. 50 µL of 10 µM Ac-ITSM(pTyr) was loaded into the syringe connected to the injection device and inserted into the sample cell. With the needle stirring at 750 rpm, 40 aliquots of 1 µL Ac-ITSM(pTyr) were injected stepwise into the sample cell with an interval of 300 seconds between each injection. The raw thermogram was converted into a binding isotherm using the MicroCal PEAQ-ITC Analysis Software (Malvern Panalytical). For the data analysis, a 1:1 binding stoichiometry between the Ac-ITSM(pTyr) and SH2 domain was assumed. ITC measurements were performed once.

6. DiFMUP phosphatase assays

SHP2 dephosphorylation of the fluorogenic substrate 6,8-difluoro-4-methylumbiliferyl phosphate (DiFMUP) was measured to evaluate the effect of acetylated peptides on SHP2 activity. Final concentrations of 0.5 nM full-length SHP2, 200 μM DiFMUP (Invitrogen) and 26 nM bisphosphorylated tandem ITIM-[dPEG4]$_2$-ITSM (CASLO ApS) were used, unless stated otherwise, per well in 96-well plates (OptiPlate-96 F Black; PerkinElmer). For peptide activation assays, each well contained 60 μL of reaction buffer (50 mM HEPES pH 7.6, 150 mM NaCl, 1 mM EDTA, 0.05% Tween 20, freshly added 5 mM DTT), 10 μL of SHP2 and 20 μL of the test peptide (0.096 nM – 51.2 μM final concentration). For peptide inhibition assays in the presence of a SHP2 activator, each well contained 40 μL of reaction buffer (50 mM HEPES pH 7.6, 150 mM NaCl, 1 mM EDTA, 0.05% Tween 20, freshly added 5 mM DTT), 10 μL of SHP2 and 40 μL of previously mixed bisphosphorylated tandem ITIM-[dPEG4]$_2$-ITSM activator and test peptide (0.096 nM – 51.2 μM or 0.376 nM – 200 μM final concentration). To obtain the maximal activity, 10 μL of SHP2 and 20 μL of bisphosphorylated tandem ITIM-[dPEG4]$_2$-ITSM activator was added to 60 μL of buffer. Samples were incubated for 20 minutes at room temperature and then 10 μL of DiFMUP was added to each sample, to give a final volume of 100 μL per well. For background subtraction, 10 μL of DiFMUP was added to 80 μL of buffer. The fluorescence was immediately measured on a Synergy H1 Microplate Reader (BioTek) with the excitation and emission monitored at 358 nm and 452 nm, respectively, over 30 minutes. Data were analysed in Excel 2016 by subtracting background values from emission values and calculating the slope of the emission profiles. Data were then further analysed in GraphPad Prism 6 by either: 1) plotting the slope against the peptide concentration and fitting the data to a variable slope model of non-linear regression; or 2) normalising the data to the maximal activity (SHP2 in the presence of ITIM-[dPEG4]$_2$-ITSM; set to 100% activity) to obtain the % activity, plotting the % activity against the peptide concentration and fitting the data to a variable slope model of non-linear regression to obtain the IC_{50} values. Data are from two or three independent experiments, each performed in technical triplicates.

7. EnzChek phosphate assays

The EnzChek Phosphate Assay Kit (Thermo Fisher Scientific), which measures the release of inorganic phosphate generated from an enzymatic reaction, was used to evaluate Ac-ITSM(pTyr) and acetylated peptide 21 as potential substrates of SHP2. A reaction mixture consisting of 43 µL of dH_2O, 0.5 µL of 1 M DTT (5 µM final concentration), 5 µL of 20X reaction buffer, 20 µL of 2-amino-6-mercapto-7-methylpurine riboside (MESG) solution, 1.5 µL of purine nucleoside phosphorylase (PNP) solution and 10 µL of 0.5 µM full-length SHP2 or PTP domain (50 nM final concentration) was added to each well in a 96-well plate (SpectraPlate-96 MB; PerkinElmer). Samples were incubated for 10 minutes at room temperature and then 20 µL of Ac-ITSM(pTyr) or peptide 21 (final concentrations: 800 µM, 400 µM, 200 µM, 100 µM, 25 µM) was added to give a final volume of 100 µL per well. The absorbance was immediately measured at 360 nm on a Synergy H1 Microplate Reader (BioTek). Data were analysed in GraphPad Prism 6 by plotting the absorbance against the peptide concentration. Data are from three independent experiments, each performed in technical triplicates.

Cellular Experiments

1. Cell culture and cell lines

Jurkat T cells were cultured in RPMI 1640 GlutaMAX (Thermo Fisher Scientific) supplemented with 10% heat-inactivated fetal bovine serum (FBS; Thermo Fisher Scientific), 100 U/mL penicillin (Sigma-Aldrich) and 100 µg/mL streptomycin (Sigma-Aldrich). HeLa cells were cultured in DMEM low glucose GlutaMAX (Thermo Fisher Scientific) supplemented with 10% fetal bovine serum (FBS; Thermo Fisher Scientific), 100 U/mL penicillin (Sigma-Aldrich) and 100 µg/mL streptomycin (Sigma-Aldrich). Both cell lines tested negative for mycoplasma contamination.

2. Peptide stability in Jurkat cell lysate

The stability of FAM-labelled peptide 26 in WT Jurkat T cell lysate was evaluated by LC-MS. 10^6 Jurkat cells were washed with 1 mL PBS and lysed in 1 mL ice-cold RIPA buffer (25 mM Tris-HCl pH 7.5, 150 mM NaCl, 1% Nonidet P-40, 1% sodium deoxycholate, 0.1% SDS) containing cOmplete EDTA-free Protease Inhibitor Cocktail (Roche), on ice for 30 minutes vortexing every 10 minutes. Cell lysate was centrifuged at 20000 x *g* for 20 minutes at 4°C. 600 µL of lysate supernatant was collected and incubated with 200 µM peptide 26 in H_2O and 200 µM FAM in DMSO for 0, 6 or 24 hours at 37°C in the dark. At each time point, 180 µL of sample was collected and proteins were precipitated by adding 45 µL of ice-cold tricholoroacetic acid (6.1 N; Sigma), vortexing 3 x 10 seconds, incubating on ice for 15 minutes and centrifuging at 20000 x *g* for 30 minutes at 4°C. The supernatant was collected on ice, filtered using a 13 mm syringe filter with a 0.2 µm PTFE membrane (Pall Life Sciences) and analysed by analytical HPLC-MS. The area under the peak at 254 nm corresponding to peptide 26 and FAM was determined. At each time point, the area under the peak of peptide 26 was normalised to that of FAM and the ratio at time point zero was set to 100%. Data are from three independent experiments.

3. Peptide immobilisation to SulfoLink beads

Cys containing peptide 27 was covalently immobilised to SulfoLink Coupling Resin (Thermo Fisher Scientific) functionalised with iodoacetyl groups. All incubations and centrifugations were performed at room temperature. Briefly, 500 µL of resin slurry (containing 250 µL of packed resin) was transferred into two microcentrifuge tubes, one for coupling to the peptide and one for the negative control. The beads were centrifuged at 1000 x *g* for 3 minutes and the supernatant discarded, then washed 3 times with 1 mL of coupling buffer (50 mM Tris pH 8.5, 5 mM EDTA-Na) for 5 minutes and the supernatant discarded each time. Peptide 27 (2 mg) in 500 µL of coupling buffer was added, while to the control 500 µL of coupling buffer alone was added, and incubated with the beads overnight. The beads were centrifuged at 1000 x *g* for 3 minutes and the supernatant discarded, then washed 3 times with 1 mL of coupling buffer for 5 minutes and the supernatant discarded each time. Any remaining binding sites on the resin were blocked by adding 500 µL of 50 mM L-cysteine hydrochloride monohydrate (Thermo Fisher Scientific) in coupling buffer and incubating for 1 hour. The beads were centrifuged at 1000 x *g* for 3 minutes and the supernatant discarded, then washed 2 times with 1 mL of coupling buffer for 5 minutes and 2 times with 1 mL of RIPA buffer (25 mM Tris-HCl pH 7.6, 150 mM NaCl, 5 mM EDTA, 1% Triton X-100, 1% sodium deoxycholate, 0.1% SDS) for 5 minutes and the supernatant discarded each time. The beads were used immediately for pulldown assays.

4. Pulldown assays using immobilised peptide

The selectivity of immobilised peptide 27 in WT Jurkat T cell lysate was determined by pulldown assays followed by mass spectrometry (MS) analysis. 2 x 10^6 Jurkat cells were washed with 20 mL PBS and lysed in 1 mL ice-cold RIPA buffer (25 mM Tris HCl pH 7.6, 150 mM NaCl, 5 mM EDTA, 1% Triton X-100, 1% sodium deoxycholate, 0.1% SDS) containing cOmplete EDTA-free Protease Inhibitor Cocktail (Roche). Samples were sonicated, incubated on ice for 30 minutes and centrifuged at 16000 x *g* for 20 minutes at 4°C. Peptide immobilised beads or negative control beads were incubated with 1 mL of Jurkat cell lysate supernatant overnight at 4°C with end-over-end rotation. Samples were centrifuged at 1000 x *g* for 5 minutes at 4°C, then the beads were washed 3 times with 1 mL of RIPA buffer for 5 minutes at 4°C. Proteins were eluted

from the beads by directly boiling in 250 µL of 2X reducing sample buffer (125 mM Tris-HCl pH 6.8, 20% glycerol, 4% SDS, 0.01% bromophenol blue, 5% β-mercaptoethanol) for 5 minutes at 95°C.

5. Mass spectrometry

The pulldown assays using immobilised peptide 27 in WT Jurkat T cell lysate were analysed by gel-enhanced liquid chromatography-mass spectrometry (LC-MS). Proteins eluted from the peptide immobilised beads and negative control beads were subjected to SDS-PAGE prior to LC-MS analysis. Proteins were separated on NuPAGE 4-12% Bis-Tris Protein Gels (Invitrogen) run in MOPS buffer (50 mM MOPS, 50 mM Tris-HCl pH 7.7, 1 mM EDTA, 0.1% SDS). The gel was stained with Colloidal Coomassie (Roth) and fractioned into 6 slices. Following reduction of Cys residues with 5 mM tris(2-carboxy-ethyl) phosphine (TCEP) dissolved in 10 mM NH_4HCO_3 (ammonium bicarbonate, AmBiC; 30 min at 56°C) and subsequent alkylation of free thiol groups with 50 mM 2-chloroacetamide (CAM) per 10 mM AmBiC (30 min at 37°C in the dark), proteins were in-gel digested using sequencing grade trypsin (Promega; 0.05 µg per slice at 37°C overnight). Peptides were desalted using C18 StageTips (Empore Octyl C18 Extraction Disks; 3M Bioanalytica), lyophilized, and reconstituted in 0.1% TFA. Reversed-phase LC-MS was performed using the UltiMateTM 3000 RSLCnano UHPLC system (Thermo Fisher Scientific) coupled to a Q Exactive Plus MS instrument (Thermo Fisher Scientific). Peptides were separated on a C18µ-precolumn (10 mm x 5 µm; µPAC 1st generation, Thermo Fisher Scientific) and a Pillar Array analytical column (500 mm x 5 µm, 100-200 Å; µPAC 1st generation, Thermo Fisher Scientific) by applying a flow rate of 300 nL/min and a 20 min linear gradient from 4-25% LC solvent B (0.1% TFA, 86% ACN) in LC solvent A (0.1% TFA) continued by a second gradient from 25-44% LC solvent B for 11 min followed by a final flush with 90% LC solvent B. The Q Exactive plus instrument was equipped with a nano-electrospray ion source (200C, 1.6-1.8 kV) and an uncoated fused silica emitter (CoAnn Technologies). Tandem mass spectrometry (MS/MS) analyses were performed in positive mode on multiply charged peptide ions. Mass spectra were acquired with a resolution of 70,000 at 200 *m/z*, a scan range of 375-1,700 *m/z*, an automatic gain control (AGC) target of 3E6 ions and a maximum ion time of 60 ms.

Multiply charged ions were selected for higher collisional dissociation (HCD) using a normalized collision energy (NCE) of 28%, fragmentation targeting for the 12 most abundant precursor ions. Fragment ions were measured with a resolution of 35,000 with a scan range of 200-2,000 *m/z* and an AGC target of 1E5. A dynamic exclusion time of 45 s with a maximum ion time of 120 ms and an isolation window of 3 *m/z* was set. Mass spectrometric data evaluation was performed using MaxQuant 1.6.5.0,[5] searching against a homo sapiens UniProt sequence database (2021/02; 218,230 entries including isoforms) supplemented with the protein sequences of the respective peptide used for the experiment. MaxQuant search was conducted using default settings, with the following exceptions: the minimum number of unique peptides a protein group have was set to 1, and the match between runs was checked. Max Quant iBAQ values were used for further analysis. The protein list was analysed with Perseus v2.0.11.[6] Missing values (NA=not available: missing) were imputed from a normal distribution using default parameters (width 0.3, down-shift 1.8, per column) and only proteins present in all samples of one experimental group were included. The data was further analysed with the R language package and identified proteins compared with the CRAPome database (*H. sapiens* single step epitope tag AP-MS background contaminant list) to mitigate the influence of background contaminants (**Table S2**).[7] For statistical analysis the limma package in R v4.1.2 was used.[8] The significance thresholds were set to alpha 0.01 and a calculated log2 protein ratio of 4. Packages used were the tidyverse v1.3.1 collection of packages, ggplot2 v3.3.5, limma 3.54.2 and ggrepel v0.9.1. Mass spectrometry data has been submitted to the ProteomeXchange Consortium (proteomecentral.proteomexchange.org) through the PRIDE partner repository under the following identifier: PXD054302.

6. Peptide cellular uptake by fluorescence microscopy

The cell permeability of FAM-labelled peptides in WT Jurkat T cells (peptide 26 and peptide 28) and WT HeLa cells (peptide 28) was visualised by fluorescence microscopy. For Jurkat cell uptake, 10^5 cells were seeded in 400 µL normal growth medium, per condition, in microcentrifuge tubes. Peptide 26 or peptide 28 (50 µM or 100 µM final concentration) was added and cells were incubated with peptide for 2 hours or 5 hours at 37°C and 5% CO_2. 8-well Lab-Tek II Chambered Coverglass

(Thermo Fisher Scientific) was coated with poly-D-lysine (poly-D-lysine hydrobromide; Sigma-Aldrich) to attach the suspension Jurkat cells. 400 µL of 0.1 mg/mL poly-D-lysine was added to each well, incubated for 5 minutes at room temperature and then aspirated. Coated wells were washed twice with 400 µL PBS and left to dry for at least 2 hours at room temperature. Cells were washed twice with 400 µL pre-warmed PBS and resuspended in 400 µL pre-warmed phenol red free RPMI 1640 (Thermo Fischer Scientific) supplemented with 10% FBS (Thermo Fisher Scientific). Cells were added to the poly-D-lysine coated wells of the chambered coverglass and allowed to adhere for 2 hours at 37°C and 5% CO_2 before imaging. For HeLa cell uptake, a 10 cm dish of cells at 90% confluency were detached and diluted 1:10 in normal growth medium. 400 µL cells were seeded per well in an 8-well Lab-Tek II Chambered Coverglass (Thermo Fisher Scientific) and allowed to adhere overnight at 37°C and 5% CO_2. Media was exchanged with 300 µL DMEM low glucose GlutaMAX (Thermo Fisher Scientific) supplemented with 0.1% FBS (Thermo Fisher Scientific) containing peptide 28 (100 µM final concentration). Cells were incubated with peptide for 30 minutes or 2 hours at 37°C and 5% CO_2. Cells were washed twice with 400 µL pre-warmed PBS and imaged in 400 µL pre-warmed phenol red free DMEM (Thermo Fisher Scientific) supplemented with 10% FBS (Thermo Fisher Scientific). Cells were imaged on an inverted confocal laser scanning microscope 880 (LSM-I-NLO; Zeiss) with fast airyscan detector (Zeiss) using the Plan-Apochromat 63x/1.4 Oil DIC M27 objective (Zeiss) and argon 488 nm laser. For Jurkat cells, a gain of 778 for the fluorescent channel and 311 for the transmission channel was used for all experiments, except for experiments with 100 µM peptide 28 where a gain of 666 for the fluorescent channel was used. For HeLa cells, a gain of 881 for the fluorescent channel was used for all experiments and the gain of the transmission channel was adjusted as required for each repeat experiment. All other microscope settings were kept constant for all experiments and images were subsequently processed using Fiji ImageJ. For Jurkat cell images, the brightness/contrast maximum was adjusted by a factor of 0.64 for the fluorescent channel and either by a factor of 0.68 or left unadjusted for the transmission channel. For HeLa cell images, the brightness/contrast maximum was adjusted to 0.3% pixel saturation for the fluorescent channel and either left unadjusted or adjusted as required for the transmission channel. Data are from three independent experiments with 3 – 5 images taken per well in each experiment (Jurkat cells), or

three or four independent experiments with 4 – 7 images taken per well in each experiment (HeLa cells).

7. Peptide cytotoxicity assays

The cytotoxicity of acetylated peptide 29 in WT Jurkat T cells and WT HeLa cells was determined using Cell Counting Kit-8 (CCK-8). Jurkat cells were seeded at 5 x 10^4 cells/well and HeLa cells were seeded at 5 x 10^3 cells/well in 96-well flat-bottom cell culture plates (Greiner Bio-One). HeLa cells were allowed to adhere overnight at 37°C and 5% CO_2. Peptide 29 was added to give a final concentration of 50 µM, 100 µM or 200 µM for Jurkat cells and 100 µM, 200 µM or 300 µM for HeLa cells in a total volume of 100 µL, each concentration in triplicate wells. In addition, three wells containing media alone as the background, three wells containing vehicle (H_2O) treated cells as the negative control and three wells containing cells treated with 5% DMSO (Jurkat cells) or 20% DMSO (HeLa cells) to induce cell death as the positive control, were included in the plate. Cells were incubated with the peptide or controls for 24 hours (Jurkat cells) or 16 hours (HeLa cells) at 37°C and 5% CO_2, then 10 µL of CCK-8 solution (Sigma-Aldrich) was added to each well and incubated for 1.5 hours at 37°C and 5% CO_2. The absorbance was measured at 460 nm on a Synergy H1 Microplate Reader (BioTek). Background values (media alone) were subtracted, negative control values (vehicle treated cells) were set to 100% and the percentage cell viability was plotted against peptide concentration in GraphPad Prism 6. Data are from three or four independent experiments, each performed in technical triplicates.

References

[1] M. Marasco, A. Berteotti, J. Weyershaeuser, N. Thorausch, J. Sikorska, J. Krausze, H. J. Brandt, J. Kirkpatrick, P. Rios, W. W. Schamel et al., *Sci. Adv.* **2020**, *6*, eaay4458.

[2] C. Meyer, M. Köhn, *Synthesis* **2011**, *2011*, 3255.

[3] J. U. Kazi, M. Vaapil, S. Agarwal, E. Bracco, S. Påhlman, L. Rönnstrand, *Cell Signal.* **2013**, *25*, 1852.

[4] C. Meyer, B. Hoeger, K. Temmerman, M. Tatarek-Nossol, V. Pogenberg, J. Bernhagen, M. Wilmanns, A. Kapurniotu, M. Köhn, *ACS Chem. Biol.* **2014**, *9*, 769.

[5] J. Cox, M. Mann, *Nat. Biotechnol.* **2008**, *26*, 1367.

[6] S. Tyanova, T. Temu, P. Sinitcyn, A. Carlson, M. Y. Hein, T. Geiger, M. Mann, J. Cox, *Nat. Methods* **2016**, *13*, 731.

[7] D. Mellacheruvu, Z. Wright, A. L. Couzens, J.-P. Lambert, N. A. St-Denis, T. Li, Y. V. Miteva, S. Hauri, M. E. Sardiu, T. Y. Low et al., *Nat. Methods* **2013**, *10*, 730.

[8] M. E. Ritchie, B. Phipson, Di Wu, Y. Hu, C. W. Law, W. Shi, G. K. Smyth, *Nucleic Acids Res.* **2015**, *43*, e47.
